# Supplementary material for: Foegraecumoside O and P, a Pair of Triterpenoid Saponins with a 4/5/6 Fused Tricyclic Oleanane Carbon Skeleton from Lysimachia foenum-graecum Hance
Source: Molecules. 2023 Jun 28;28(13):5061. doi: 10.3390/molecules28135061 (PMC10343785; doi:10.3390/molecules28135061)
Supplement: Supplementary file 1 [file molecules-28-05061-s001.zip › molecules-2461665-supplementary.pdf]

Supplementary data

Foegraecumoside O and P, a Pair of Triterpenoid Saponins with a 4/5/6 Fused Tricyclic Oleanane Carbon Skeleton from *Lysimachia foenum-graecum* Hance

Lumei Dai [1,2,†], Shuang He [1,†], Bin Zhang [1], Hengshan Wang [1], Yan Wang [1,3,*] and Dong Liang [1,*]

[1] State Key Laboratory for Chemistry and Molecular Engineering of Medicinal Resources, School of Chemistry and Pharmaceutical Sciences, Guangxi Normal University, Guilin-541004, People's Republic of China; dlmei610@163.com (L.M.D.); m15077301367@163.com (S.H.); zhbin308@163.com (B.Z.), whengshan@163.com (H.S.W.).

[2] School of Biological and Food Engineering, Huanghuai University, Zhumadian-463000, People's Republic of China

[3] H. E. J. Research Institute of Chemistry, International Center for Chemical and Biological Sciences, University of Karachi, Karachi-75270, Pakistan

* Correspondence: liangdonggxnu@163.com (D.L.); yan.wang@iccs.edu (Y.W.)

† These authors contributed equally to this work.

Table of Contents

Figure S1. IR spectrum of compound **1** 4
Figure S2. HR-ESI-MS spectrum of compound **1** 4
Figure S3. ^{1}H NMR spectrum of compound **1** (pyridine-d$_5$, 500 MHz) 5
Figure S4. ^{1}H NMR assignment-1 of compound **1** 5
Figure S5. ^{1}H NMR assignment-2 of compound **1** 6
Figure S6. ^{1}H NMR assignment-3 of compound **1** 6
Figure S7. ^{13}C NMR spectrum of compound **1** (pyridine-d$_5$, 125 MHz) 7
Figure S8. ^{13}C NMR assignment-1 of compound **1** 7
Figure S9. ^{13}C NMR assignment-2 of compound **1** 8
Figure S10. DEPT spectrum of compound **1** 9
Figure S11. COSY spectrum-1 of compound **1** 9
Figure S12. COSY spectrum-2 of compound **1** 10
Figure S13. COSY spectrum-3 of compound **1** 10
Figure S14. HSQC spectrum-1 of compound **1** 11
Figure S15. HSQC spectrum-2 of compound **1** 11
Figure S16. HSQC spectrum-3 of compound **1** 12
Figure S17. HMBC spectrum-1 of compound **1** 12
Figure S18. HMBC spectrum-2 of compound **1** 13
Figure S19. HMBC spectrum-3 of compound **1** 13
Figure S20. NOESY spectrum-1 of compound **1** 14
Figure S21. NOESY spectrum-2 of compound **1** 14
Figure S22. NOESY spectrum-3 of compound **1** 14
Figure S23. IR spectrum of compound **2** 15
Figure S24. HR-ESI-MS spectrum of compound **2** 15
Figure S25. ^{1}H NMR spectrum of compound **2** (pyridine-d$_5$, 600 MHz) 16
Figure S26. ^{1}H NMR assignment-1 of compound **2** 16
Figure S27. ^{1}H NMR assignment-2 of compound **2** 17
Figure S28. ^{1}H NMR assignment-3 of compound **2** 17
Figure S29. ^{13}C NMR spectrum of compound **2** (pyridine-d$_5$, 150 MHz) 18
Figure S30. ^{13}C NMR assignment-1 of compound **2** 18
Figure S31. ^{13}C NMR assignment-2 of compound **2** 19
Figure S32. ^{13}C NMR assignment-3 of compound **2** 19
Figure S33. COSY spectrum-1 of compound **2** 20
Figure S34. COSY spectrum-2 of compound **2** 20
Figure S35. COSY spectrum-3 of compound **2** 21
Figure S36. HSQC spectrum-1 of compound **2** 21
Figure S37. HSQC spectrum-2 of compound **2** 22
Figure S38. HSQC spectrum-3 of compound **2** 22
Figure S39. HMBC spectrum-1 of compound **2** 23
Figure S40. HMBC spectrum-2 of compound **2** 23
Figure S41. HMBC spectrum-3 of compound **2** 24
Figure S42. NOESY spectrum-1 of compound **2** 24
Figure S43. NOESY spectrum-2 of compound **2** 25

Table S1. Experimental and computed ^{13}C NMR chemical shifts of **1** and **2** 25
Table S2. Experimental and computed ^{1}H NMR chemical shifts of **1** and **2** 26
Table S3. Statistics of ordinary least squares (OLS) linear regression of experimental and computed ^{13}C and ^{1}H NMR chemical shifts of **1** and **2** 27
Figure S44. Isolation and purification of compounds **1** and **2** 28

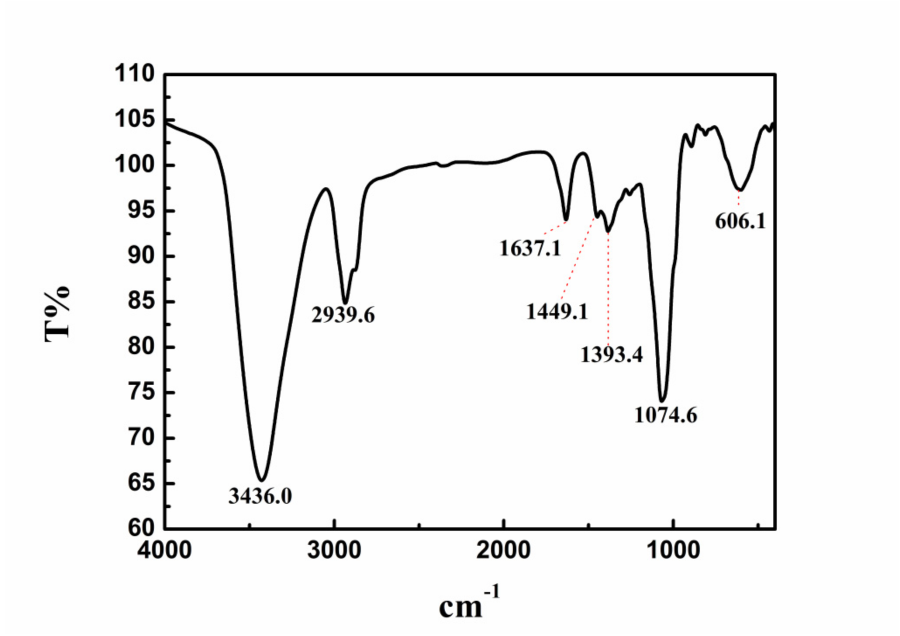

Figure S1. IR spectrum of compound **1**

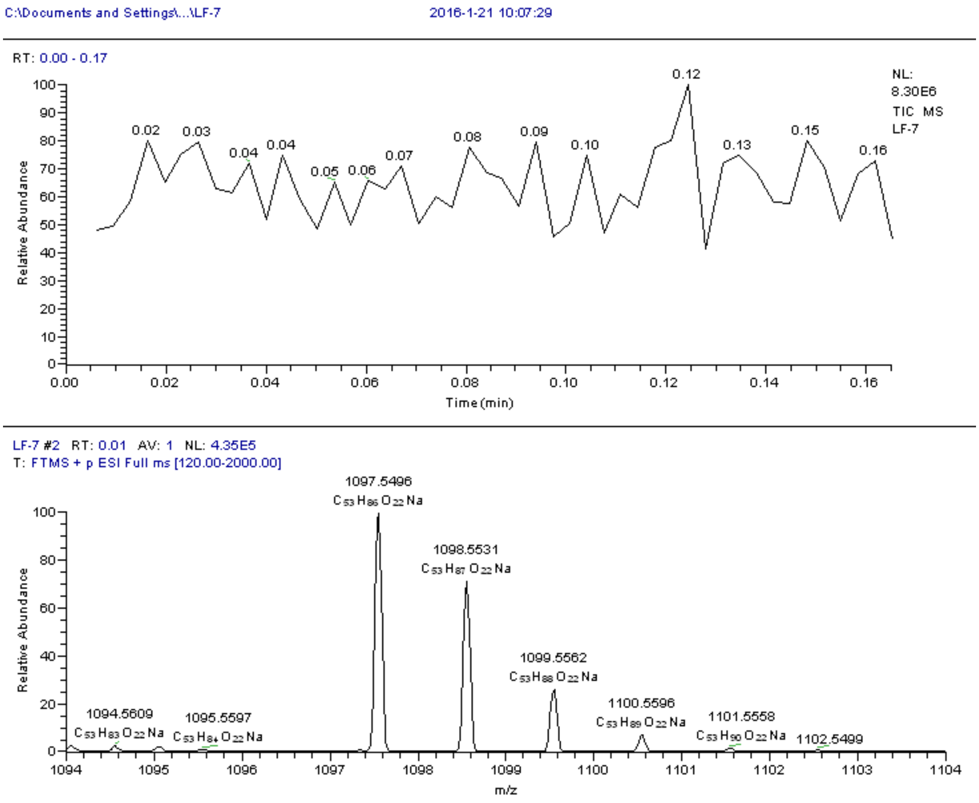

Figure S2. HR-ESI-MS spectrum of compound **1**

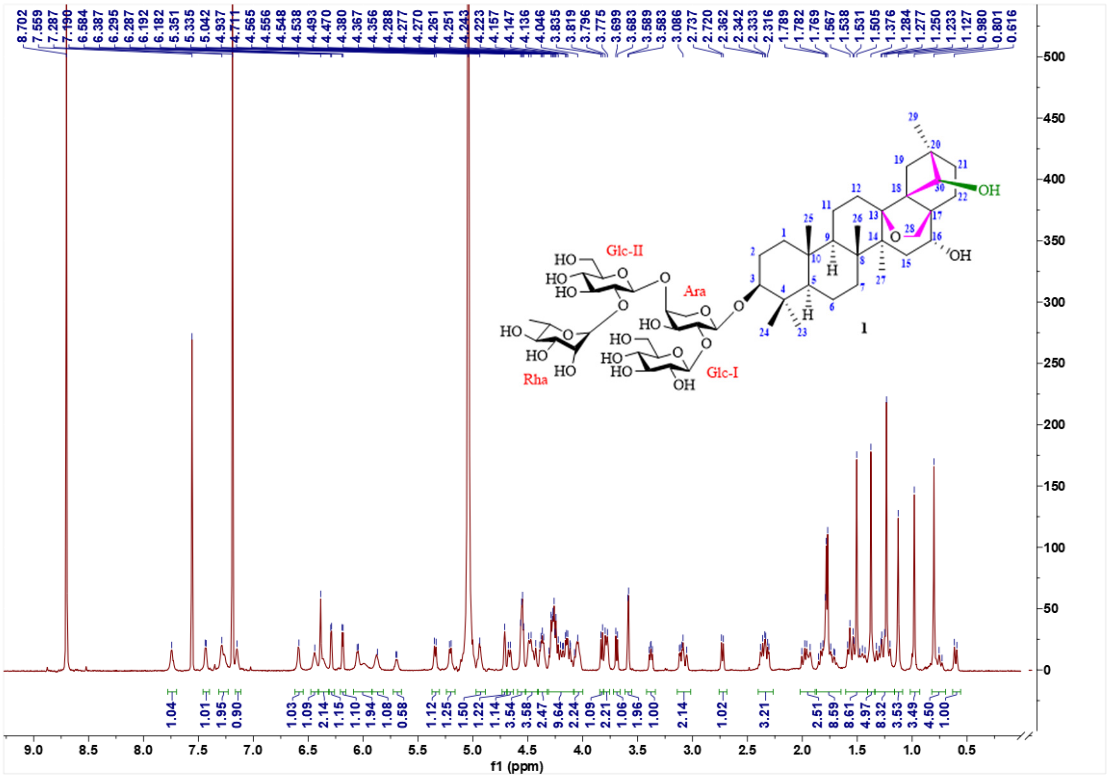

Figure S3. ^{1}H NMR spectrum of compound **1** (pyridine-d_5, 500 MHz)

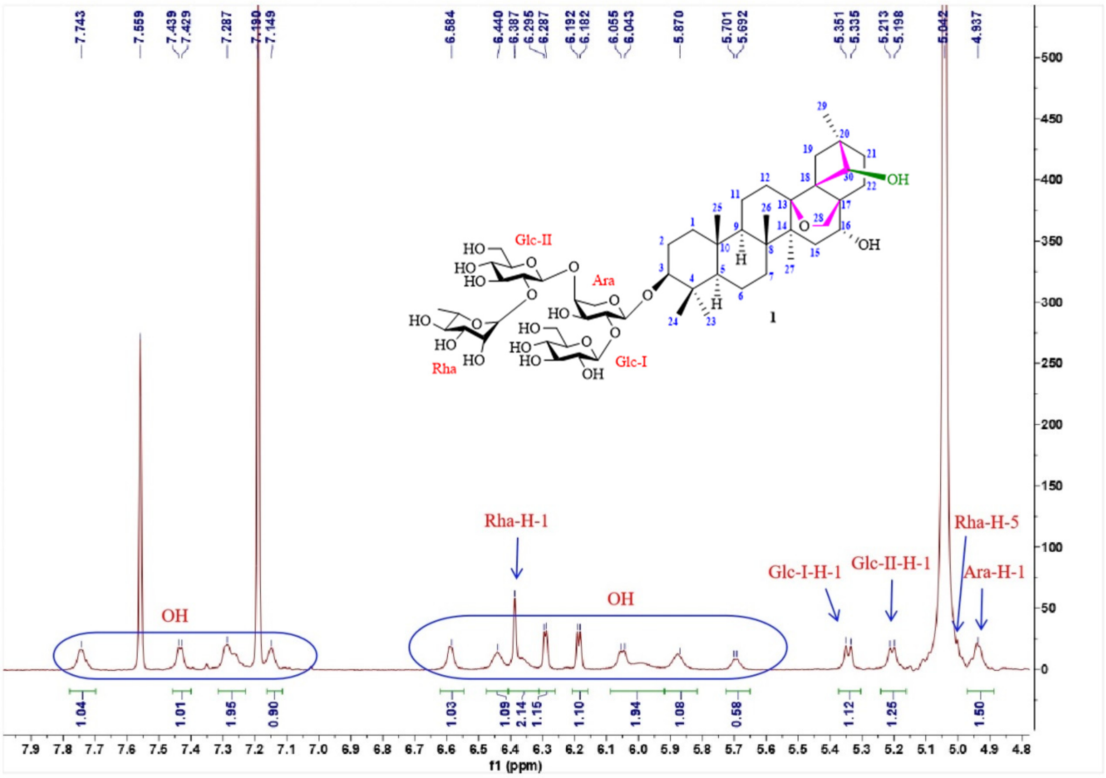

Figure S4. ^{1}H NMR assignment-1 of compound **1**

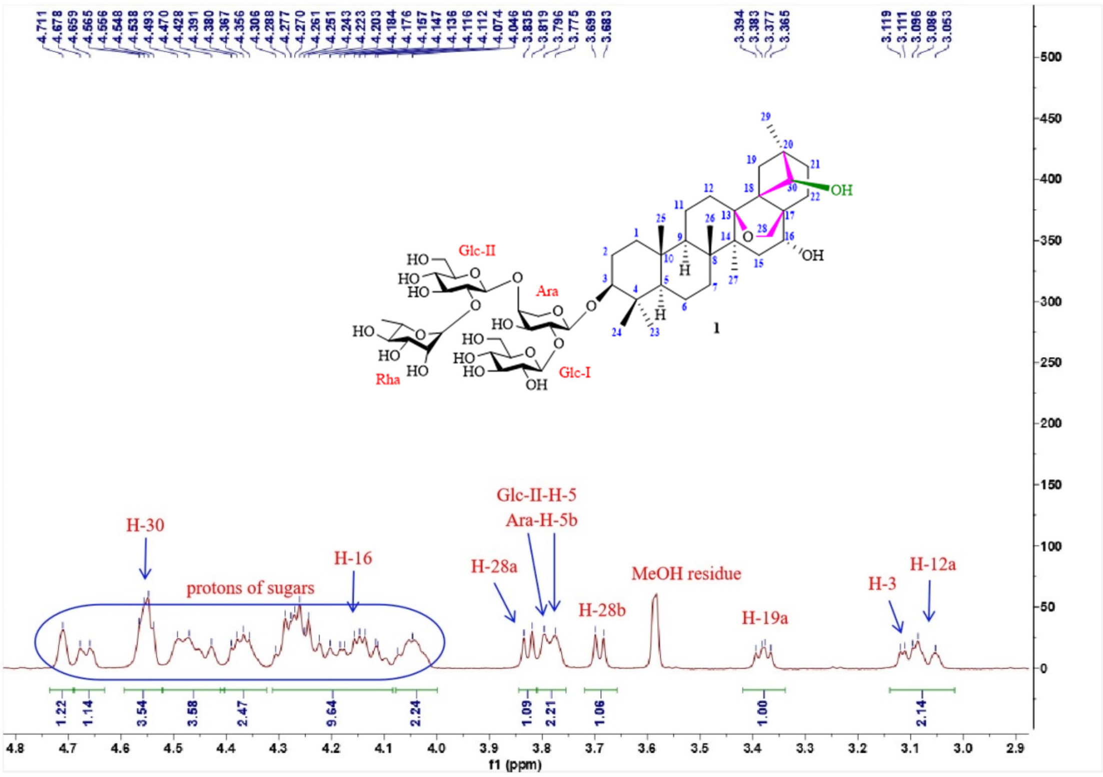

Figure S5. ^{1}H NMR assignment-2 of compound **1**

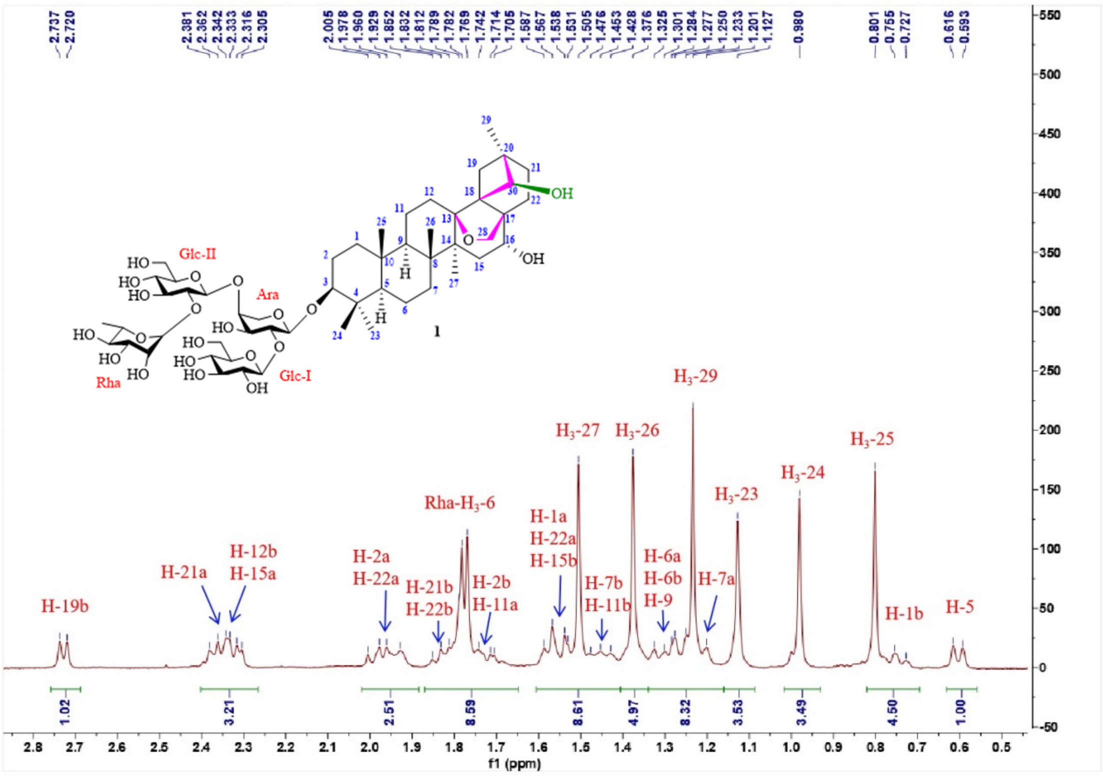

Figure S6. ^{1}H NMR assignment-3 of compound **1**

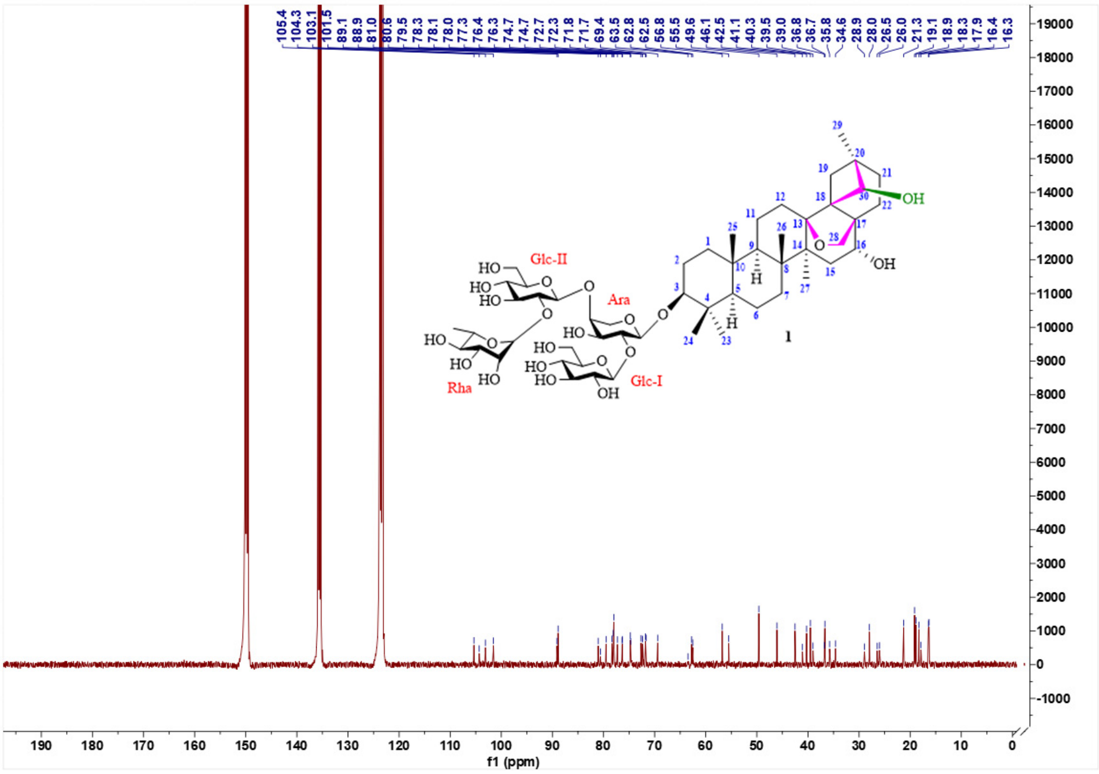

Figure S7. ^{13}C NMR spectrum of compound **1** (pyridine-d_5, 125 MHz)

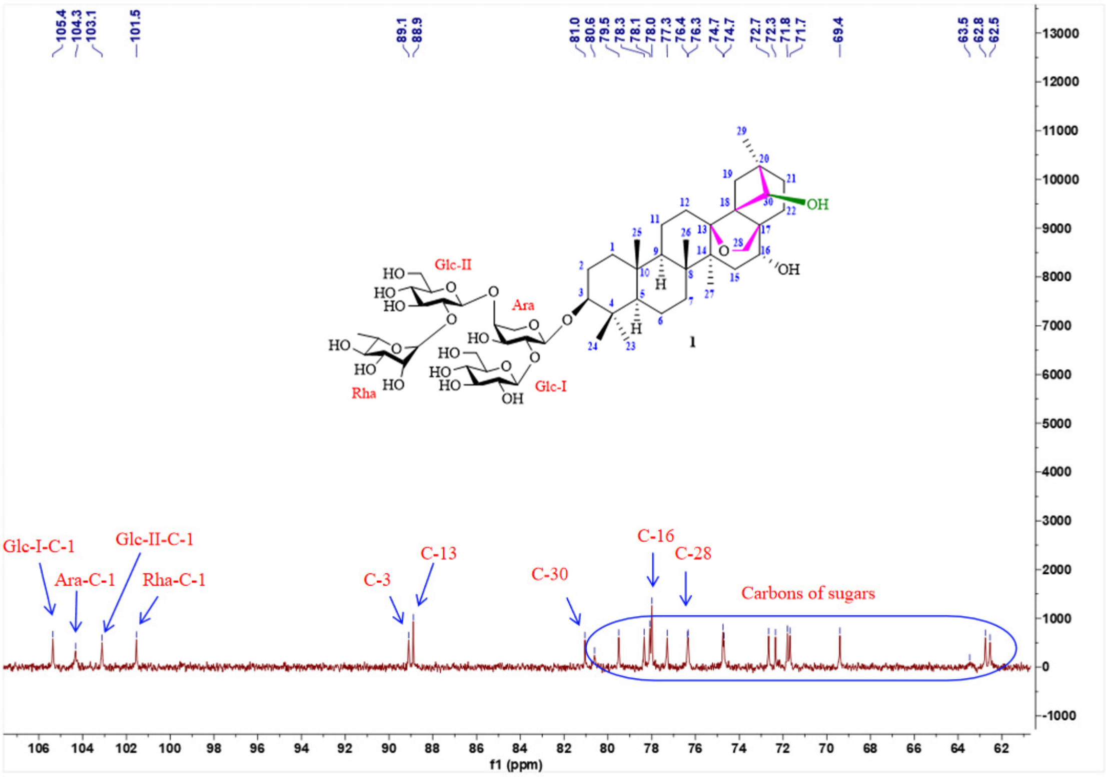

Figure S8. ^{13}C NMR assignment-1 of compound **1**

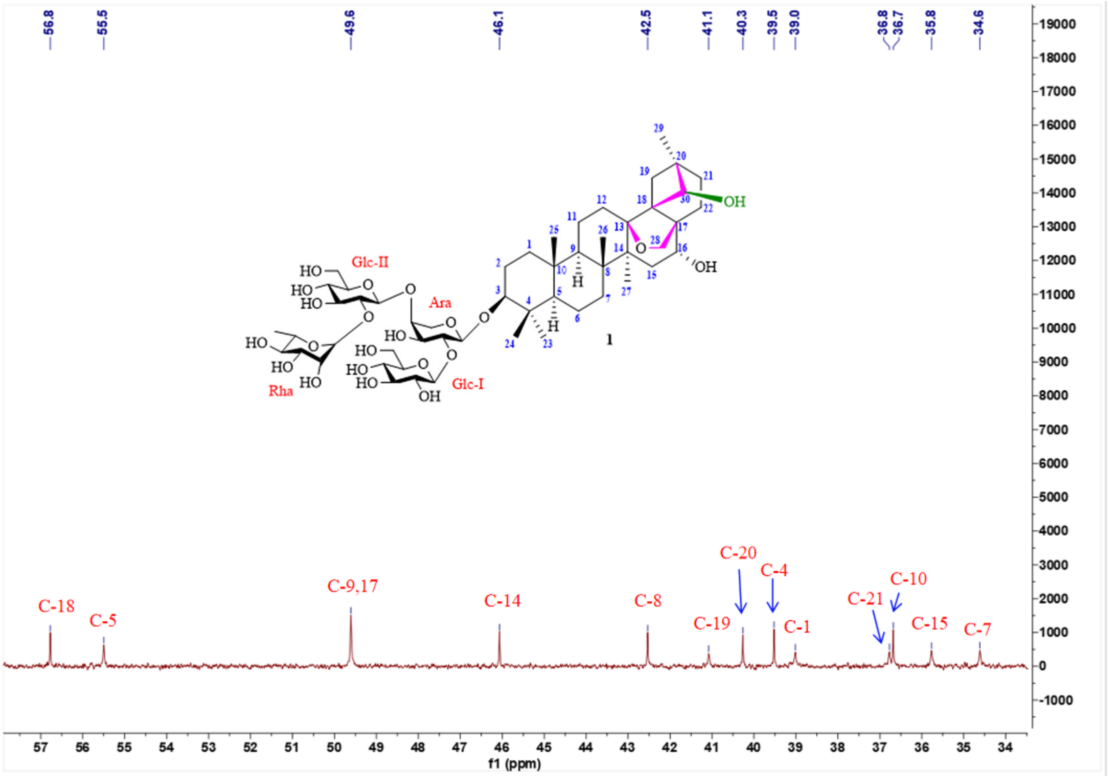

Figure S9. ^{13}C NMR assignment-2 of compound **1**

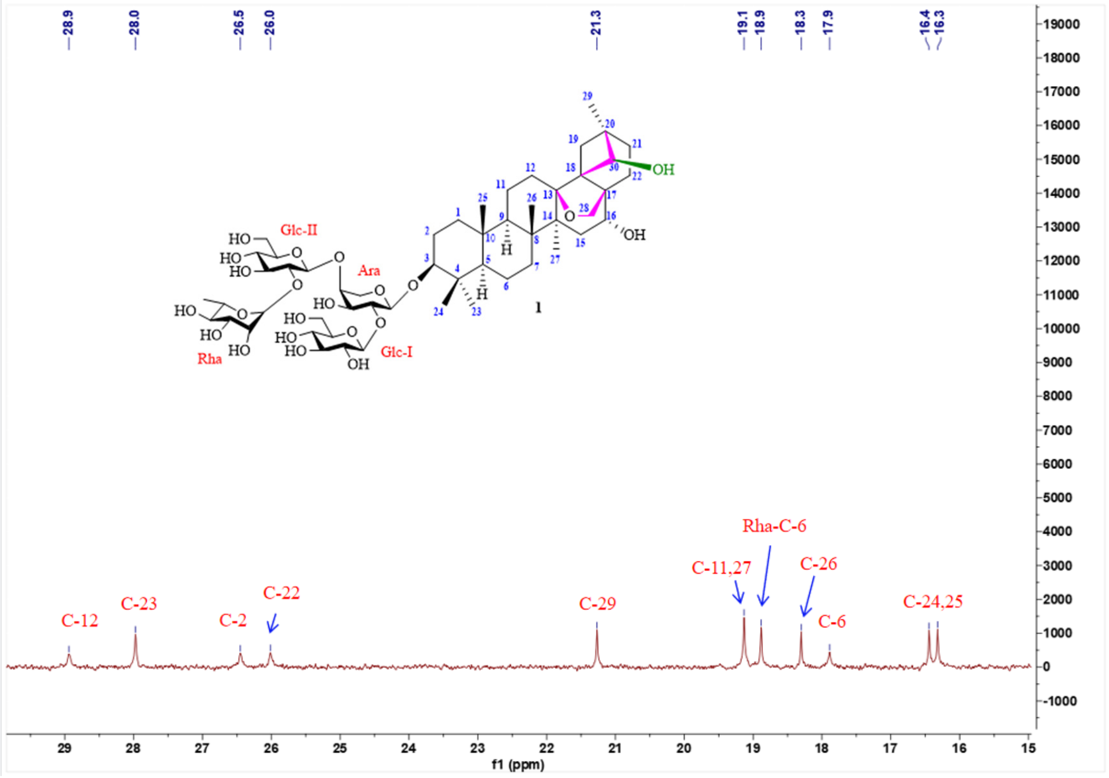

Figure S10. ^{13}C NMR assignment-3 of compound **1**

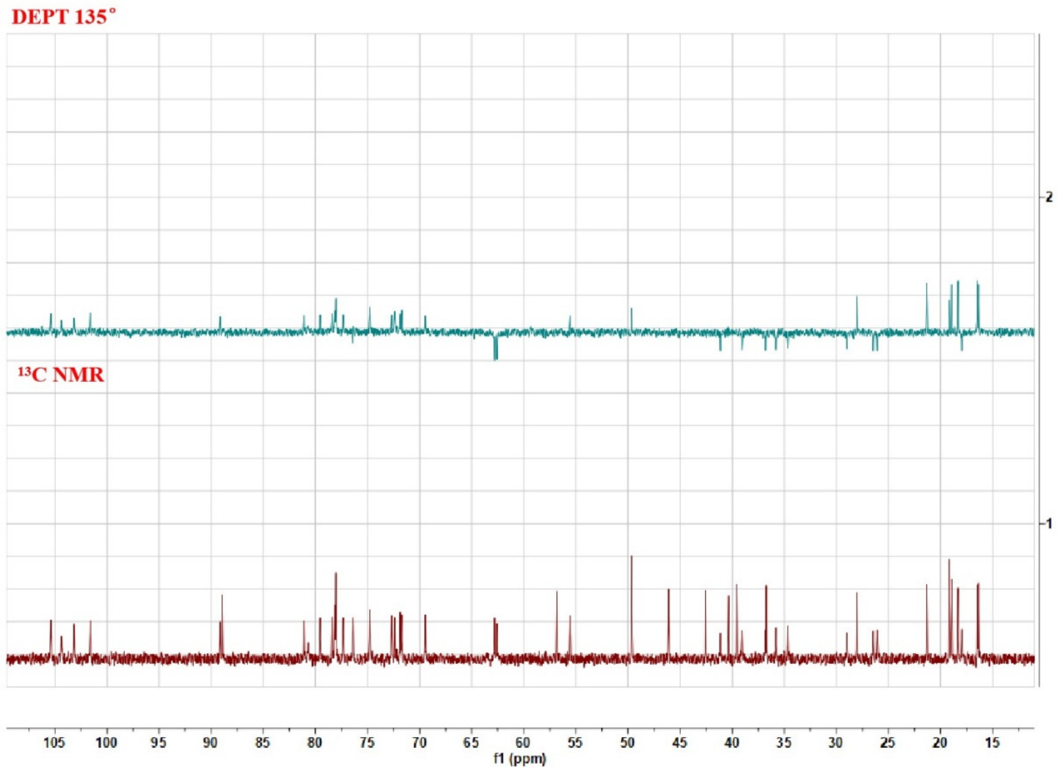

Figure S11. DEPT spectrum of compound **1**

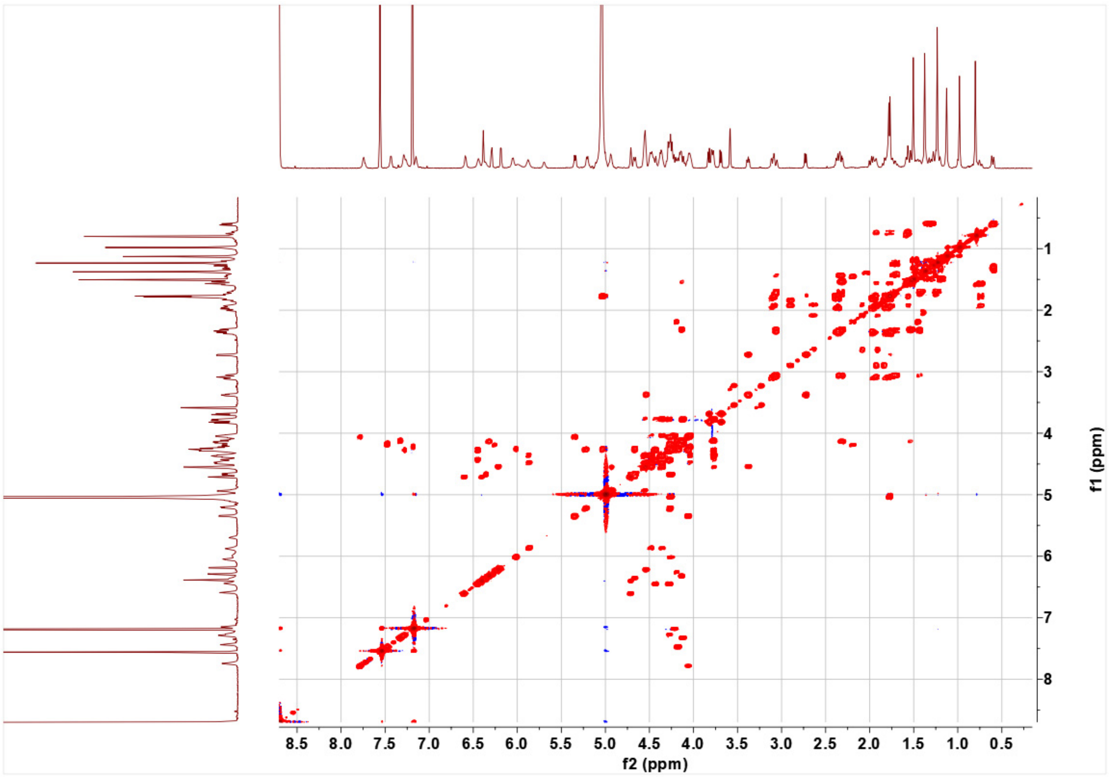

Figure S12. COSY spectrum-1 of compound **1**

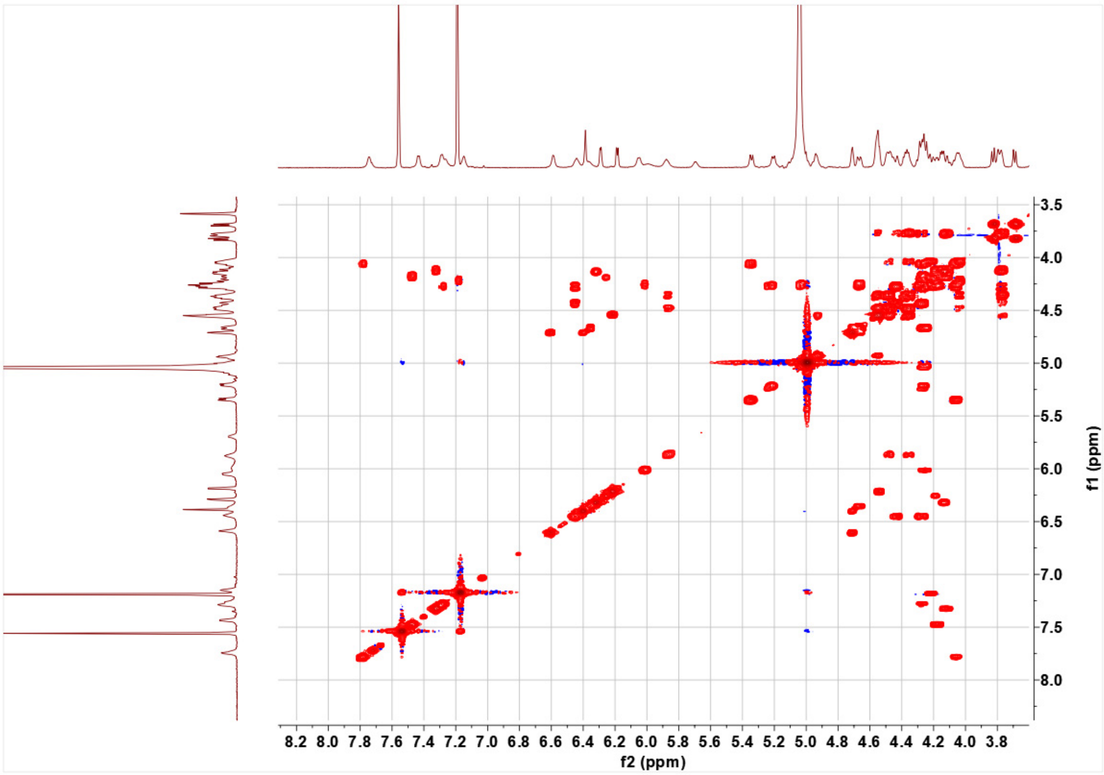

Figure S13. COSY spectrum-2 of compound **1**

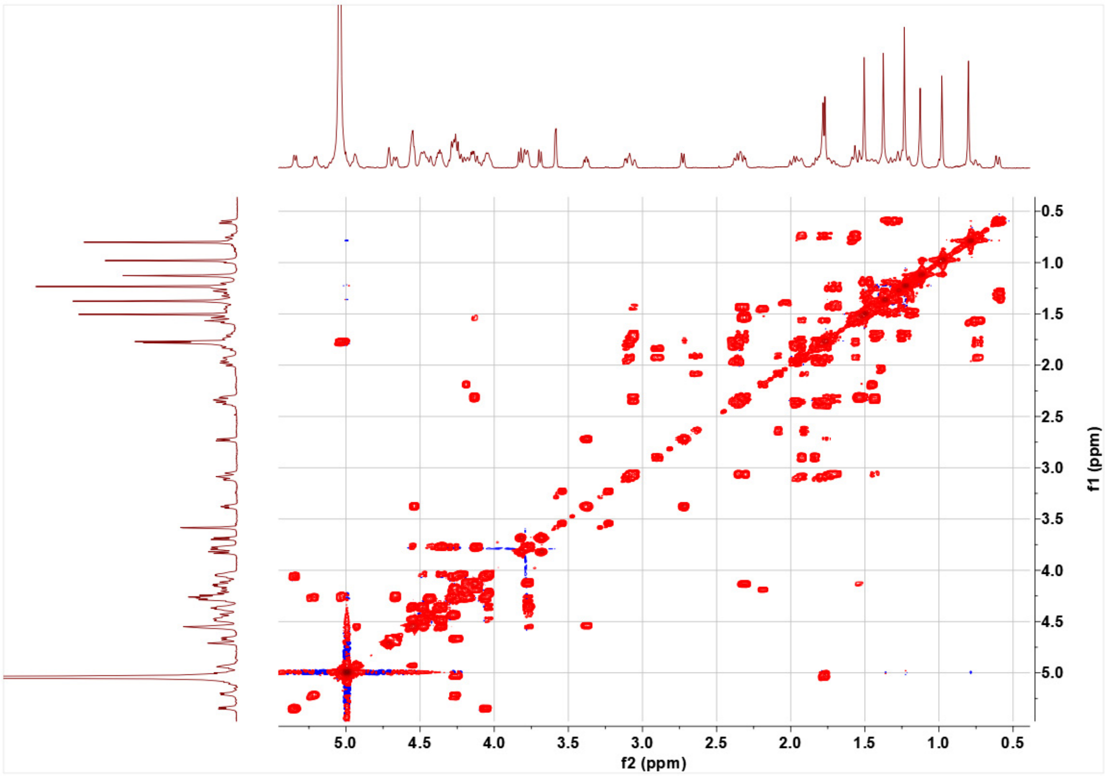

Figure S14. COSY spectrum-3 of compound **1**

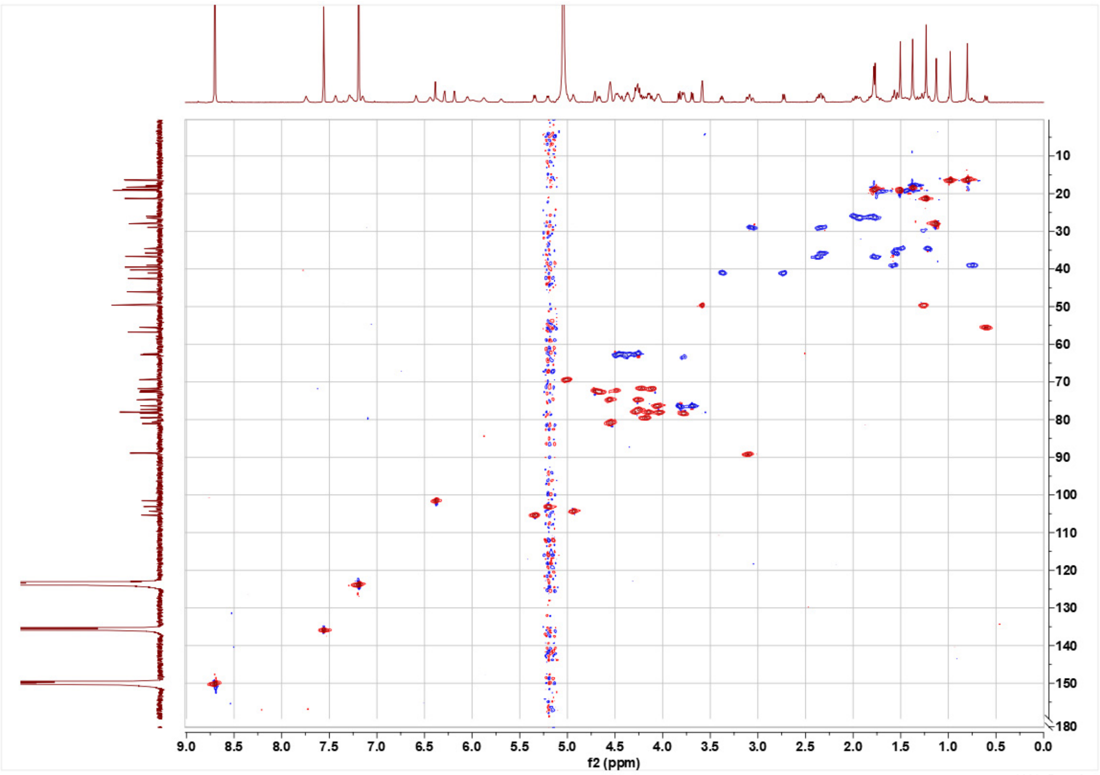

Figure S15. HSQC spectrum-1 of compound **1**

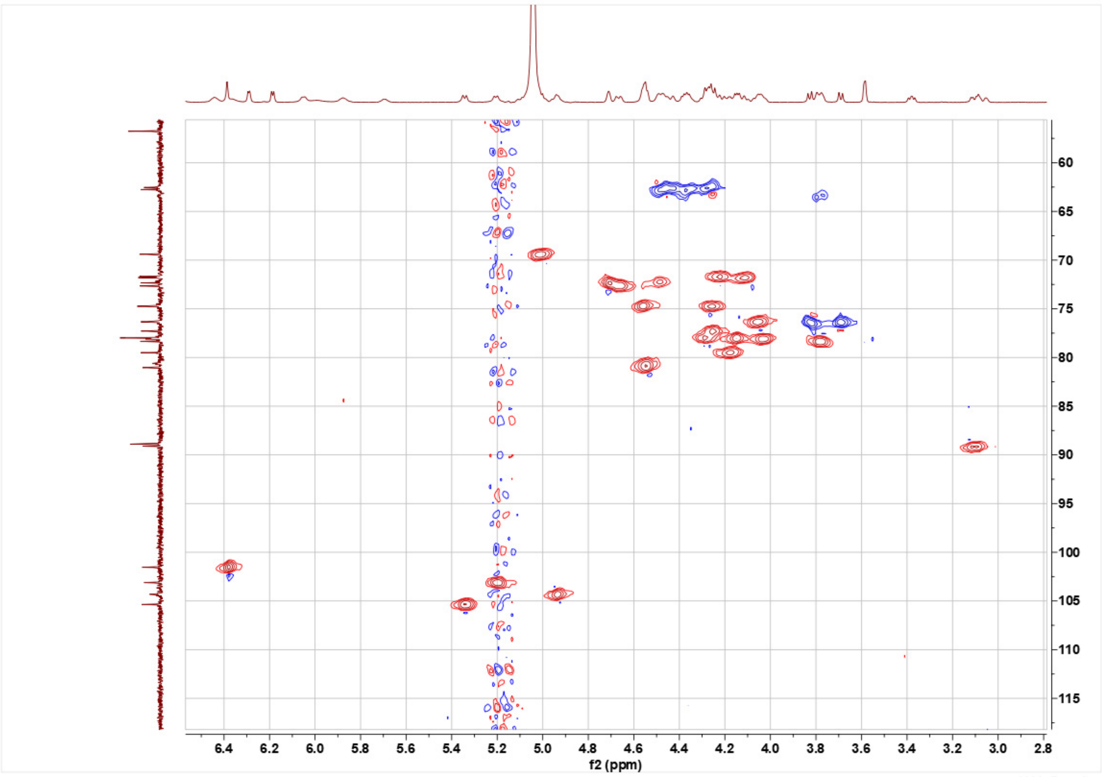

Figure S16. HSQC spectrum-2 of compound **1**

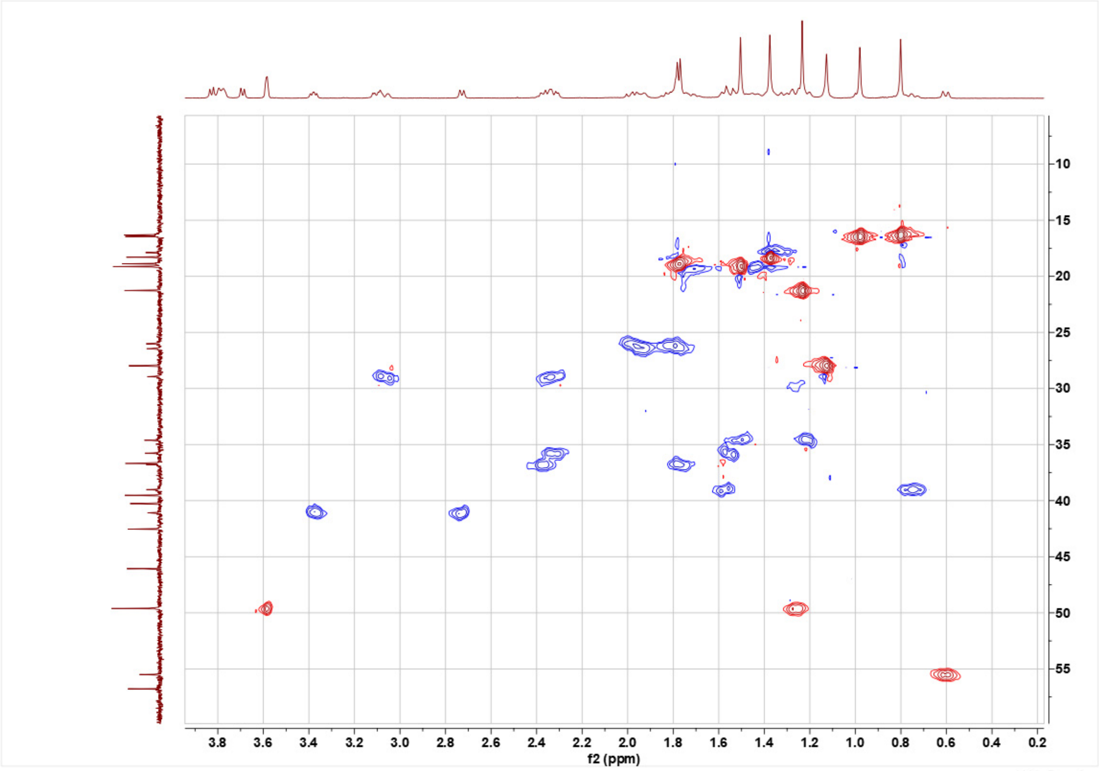

Figure S17. HSQC spectrum-3 of compound **1**

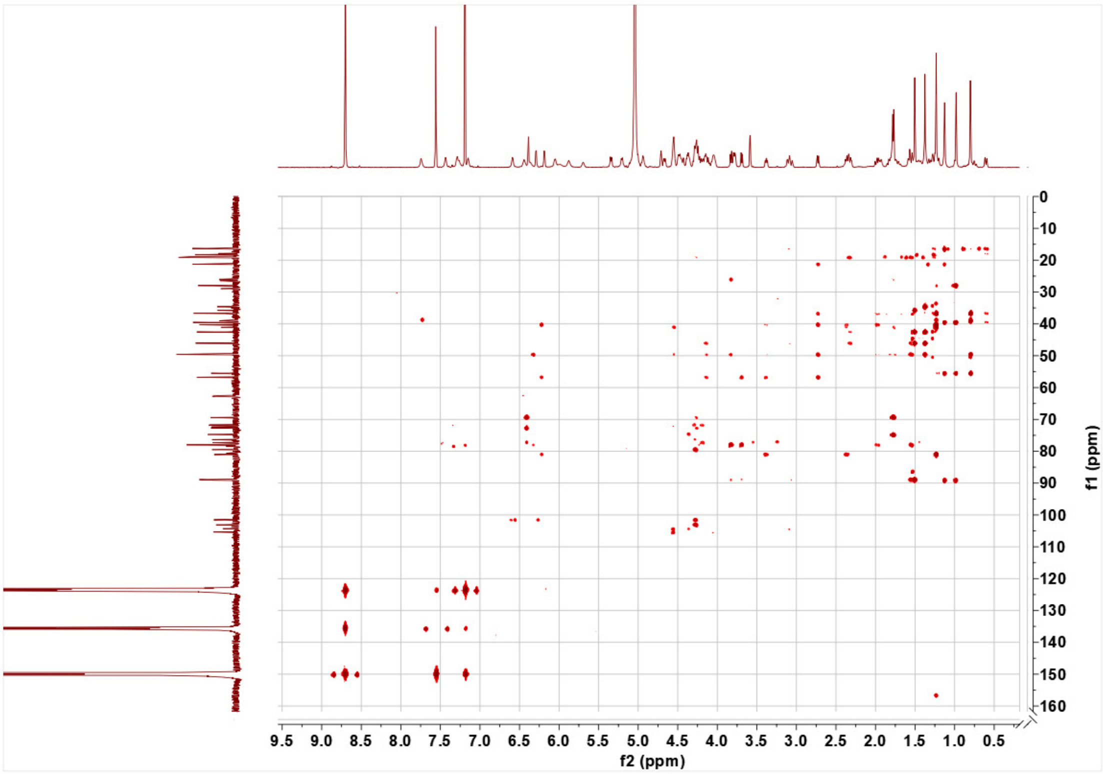

Figure S18. HMBC spectrum-1 of compound **1**

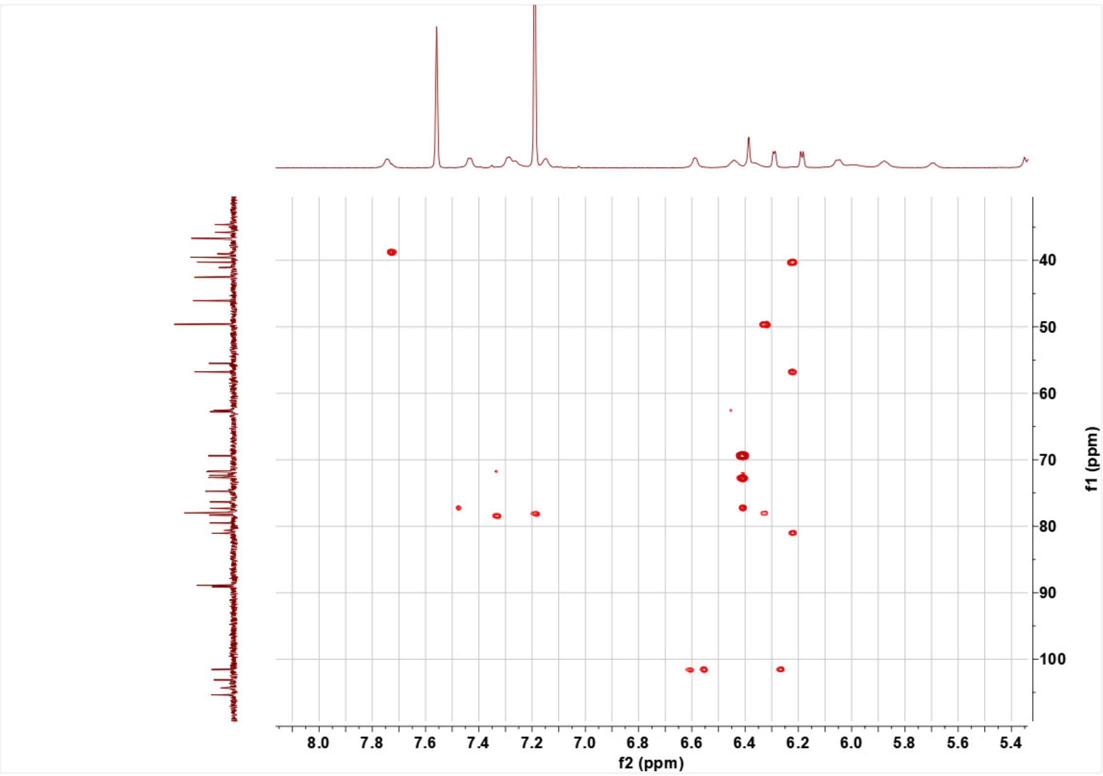

Figure S19. HMBC spectrum-2 of compound **1**

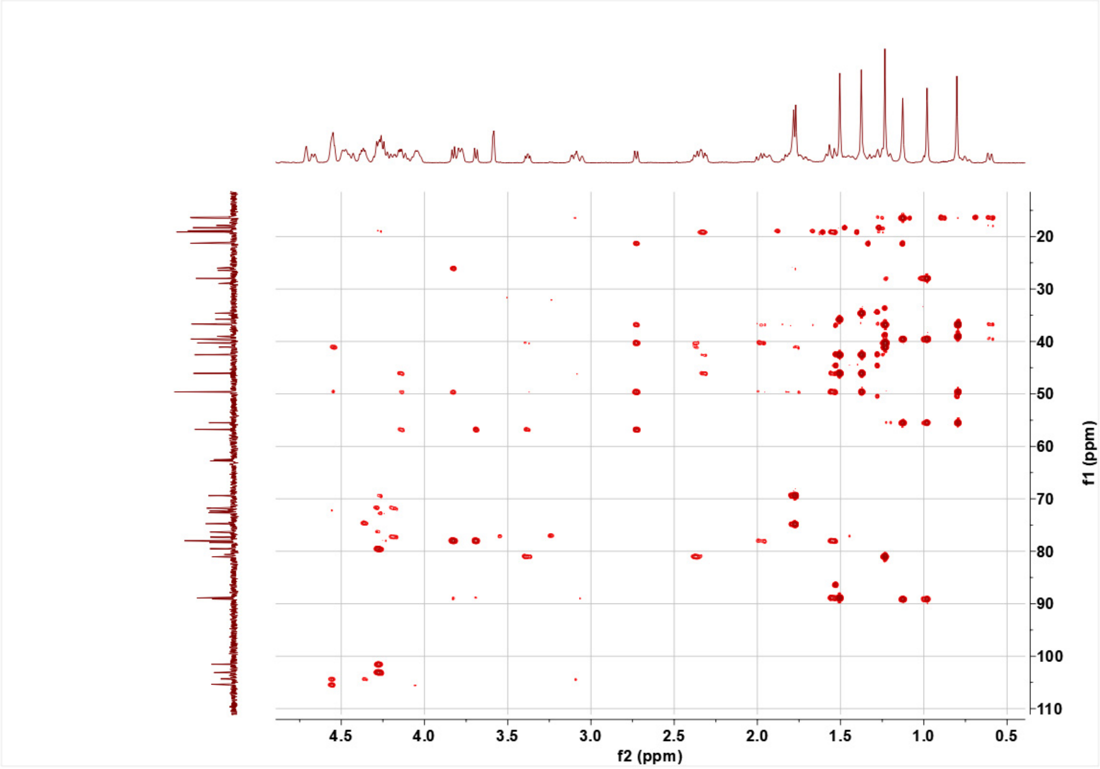

Figure S20. HMBC spectrum-3 of compound **1**

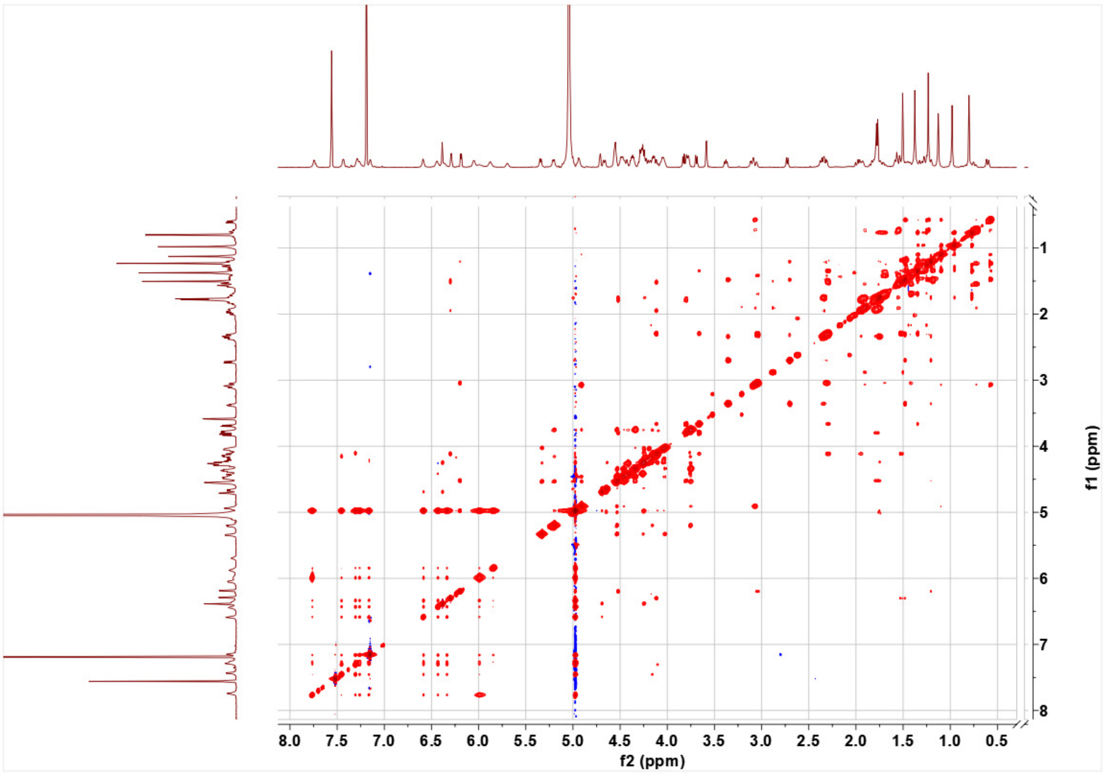

Figure S21. NOESY spectrum-1 of compound **1**

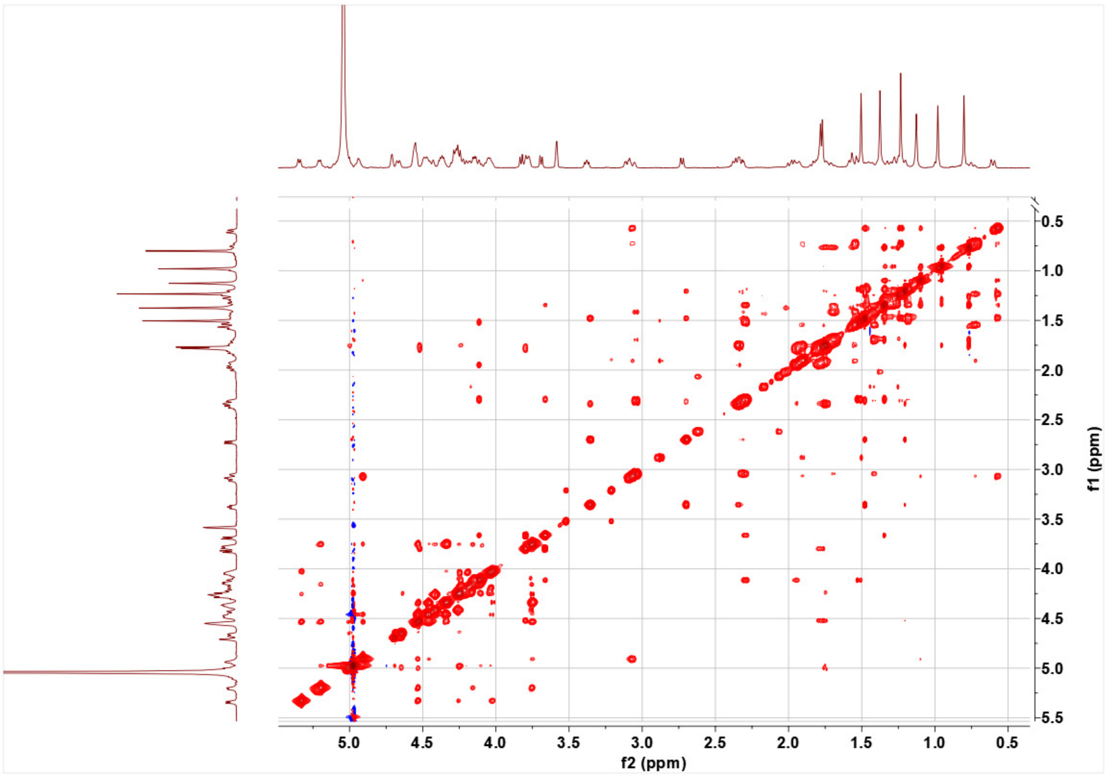

Figure S22. NOESY spectrum-2 of compound **1**

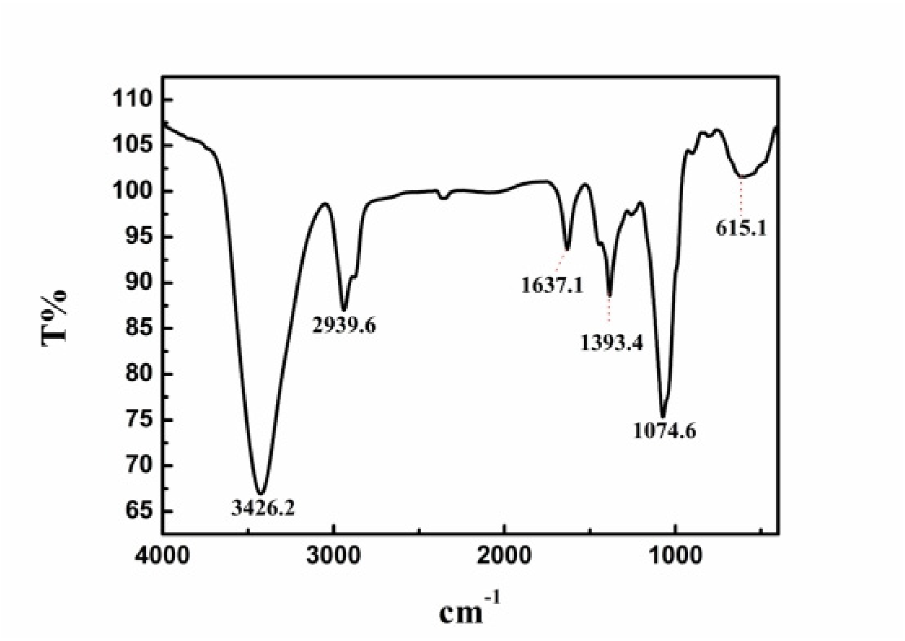

Figure S23. IR spectrum of compound **2**

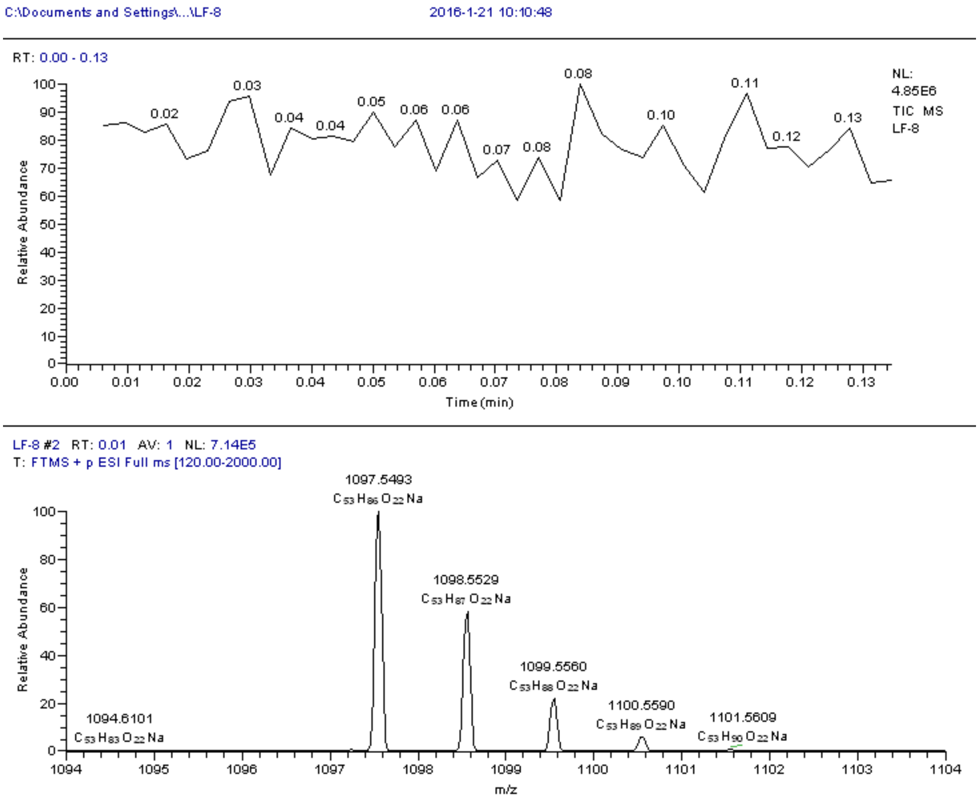

Figure S24. HR-ESI-MS spectrum of compound **2**

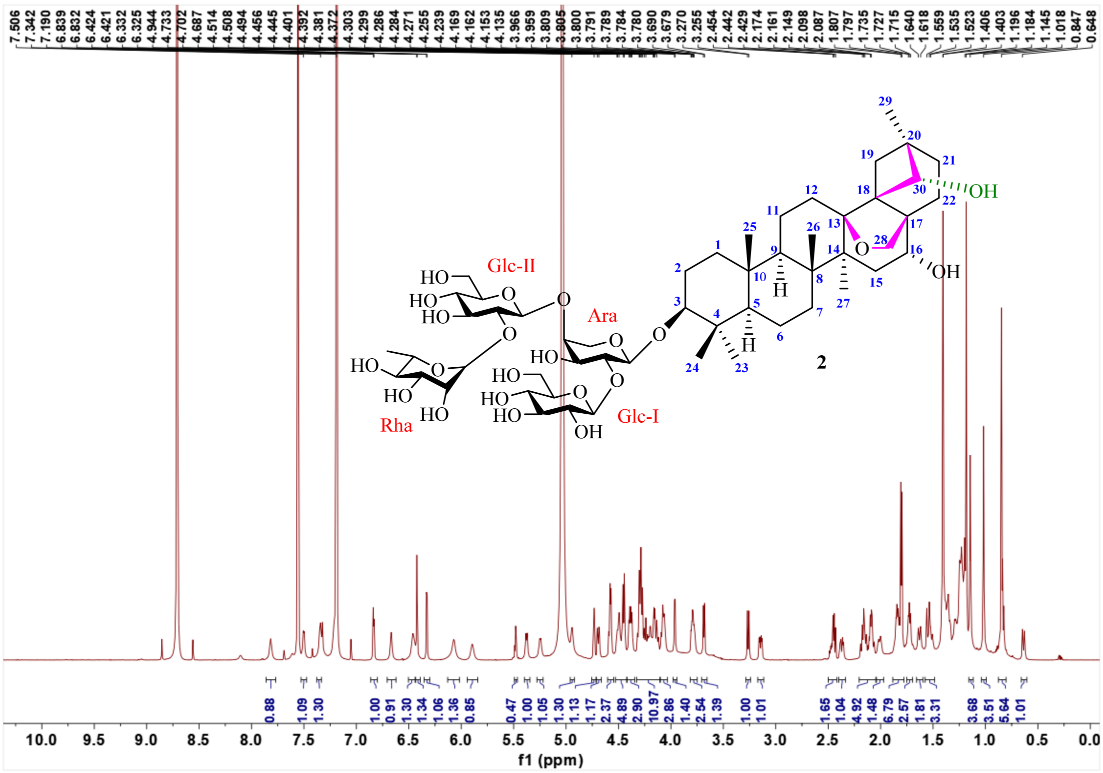

Figure S25. ^{1}H NMR spectrum of compound **2** (pyridine-d5, 600 MHz)

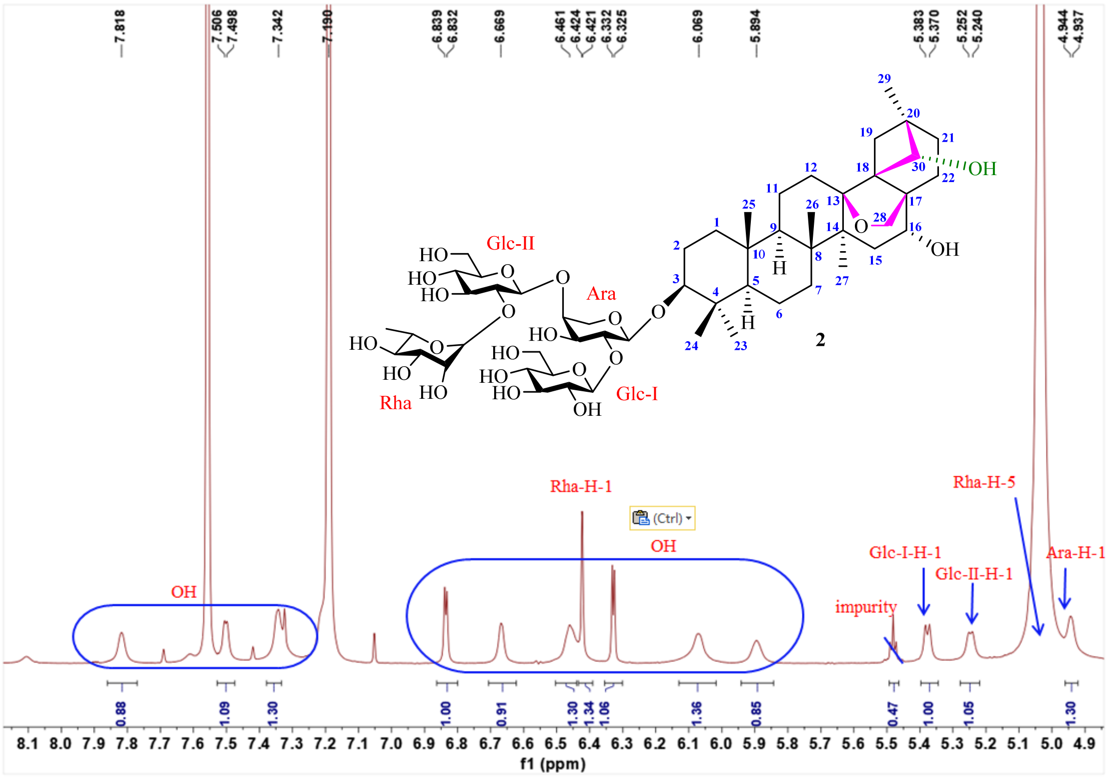

Figure S26. ^{1}H NMR assignment-1 of compound **2**

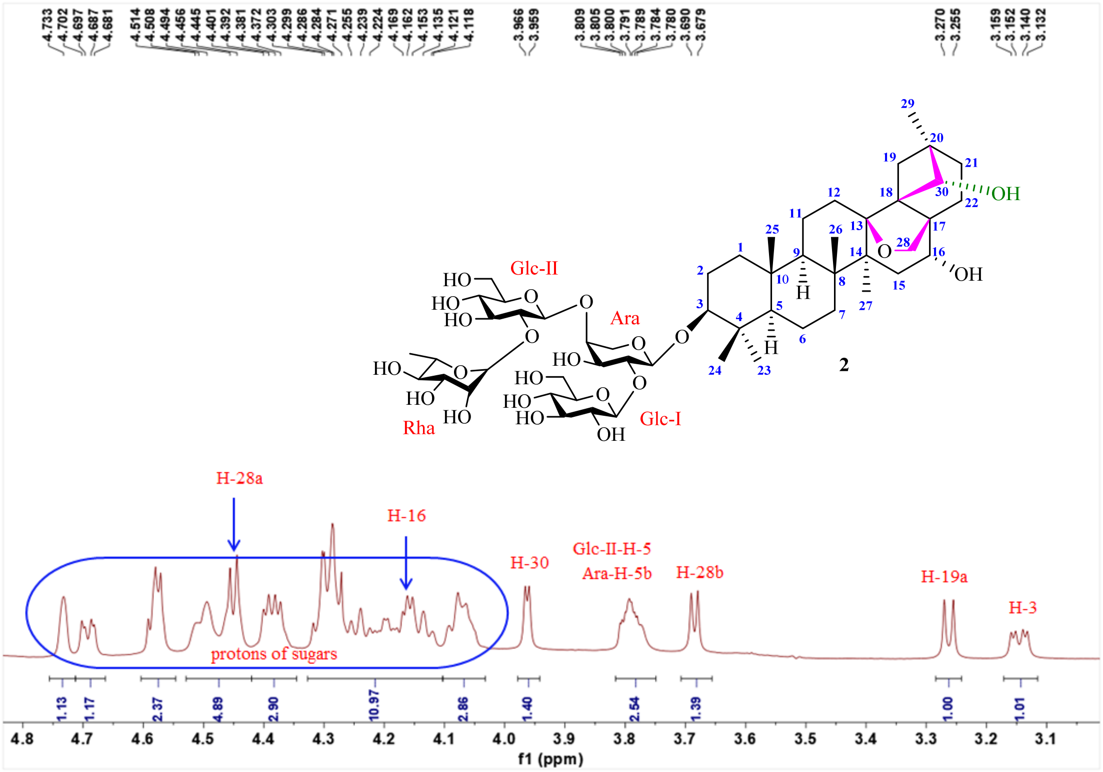

Figure S27. ^{1}H NMR assignment-2 of compound **2**

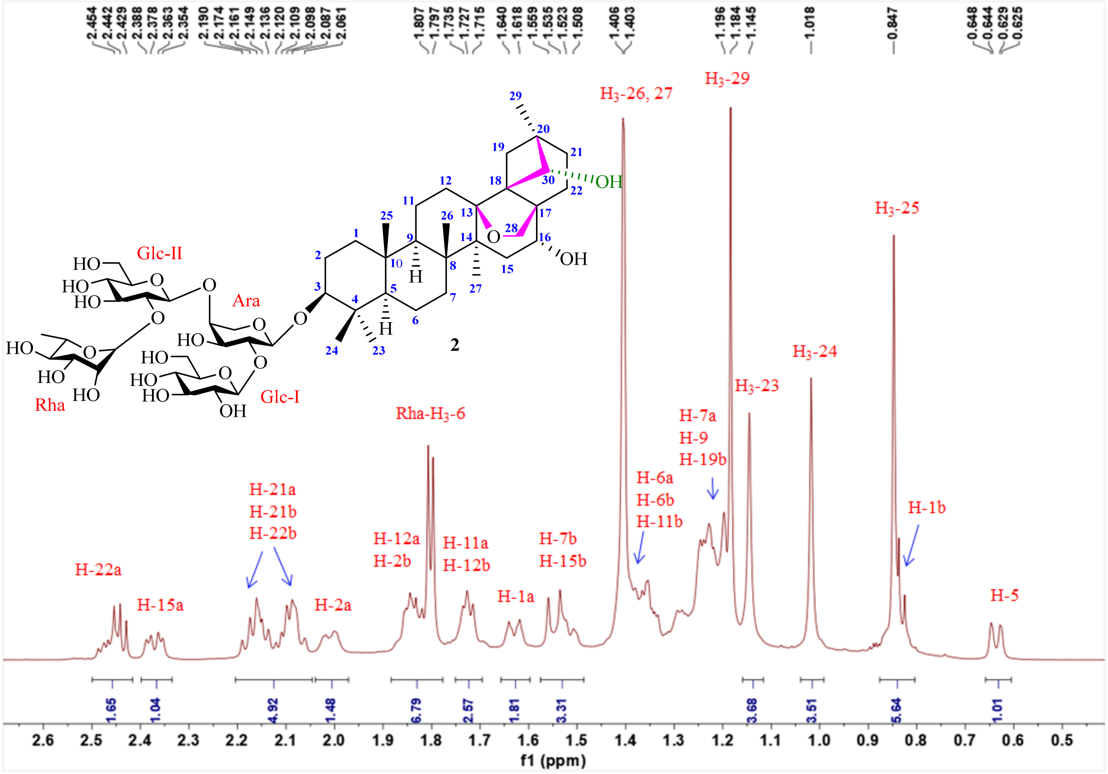

Figure S28. ^{1}H NMR assignment-3 of compound **2**

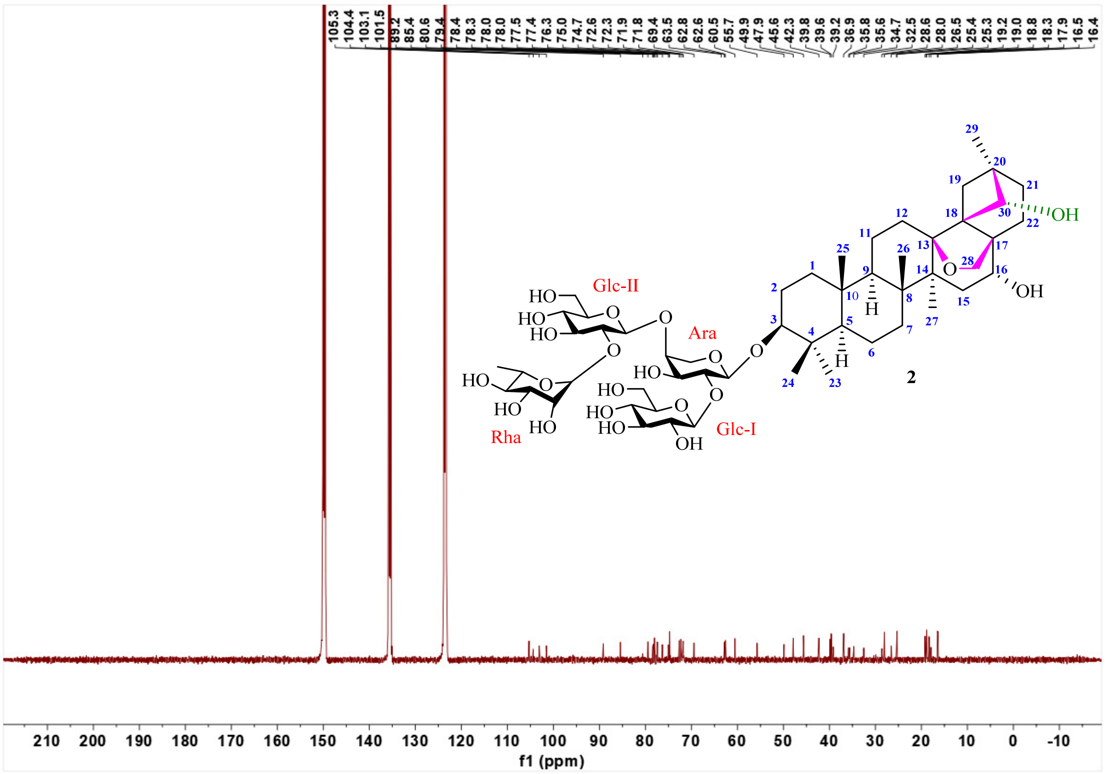

Figure S29. ^{13}C NMR spectrum of compound **2** (pyridine-d_5, 150 MHz)

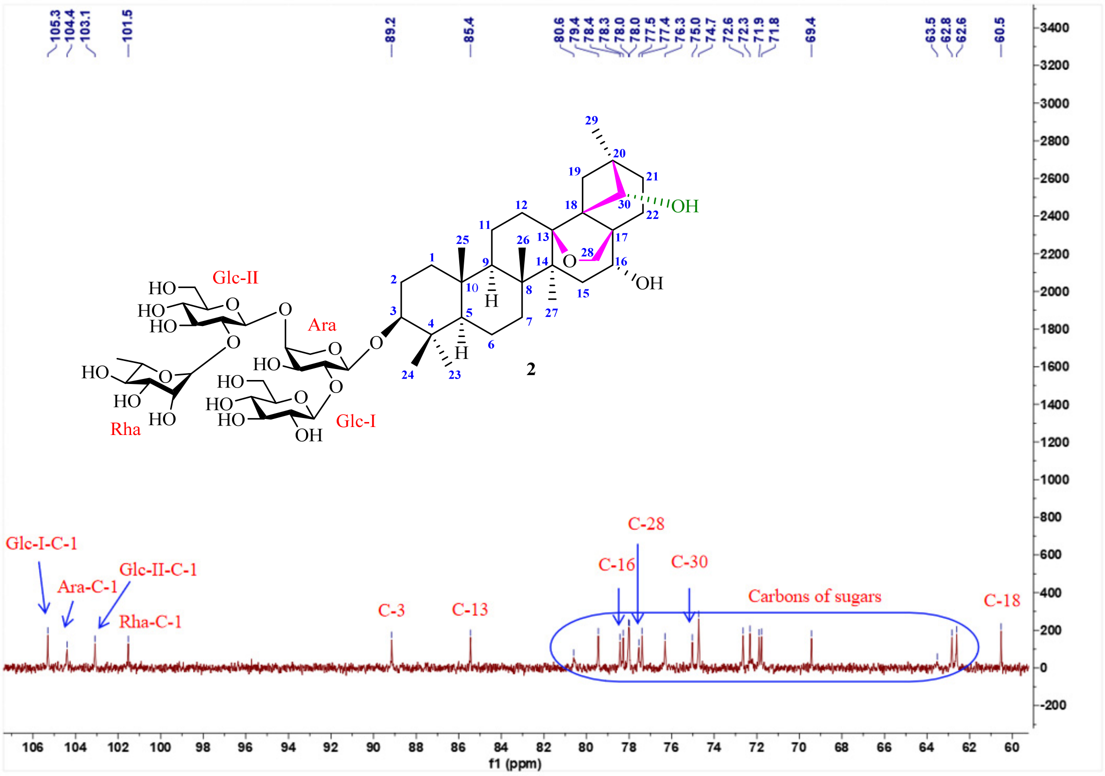

Figure S30. ^{13}C NMR assignment-1 of compound **2**

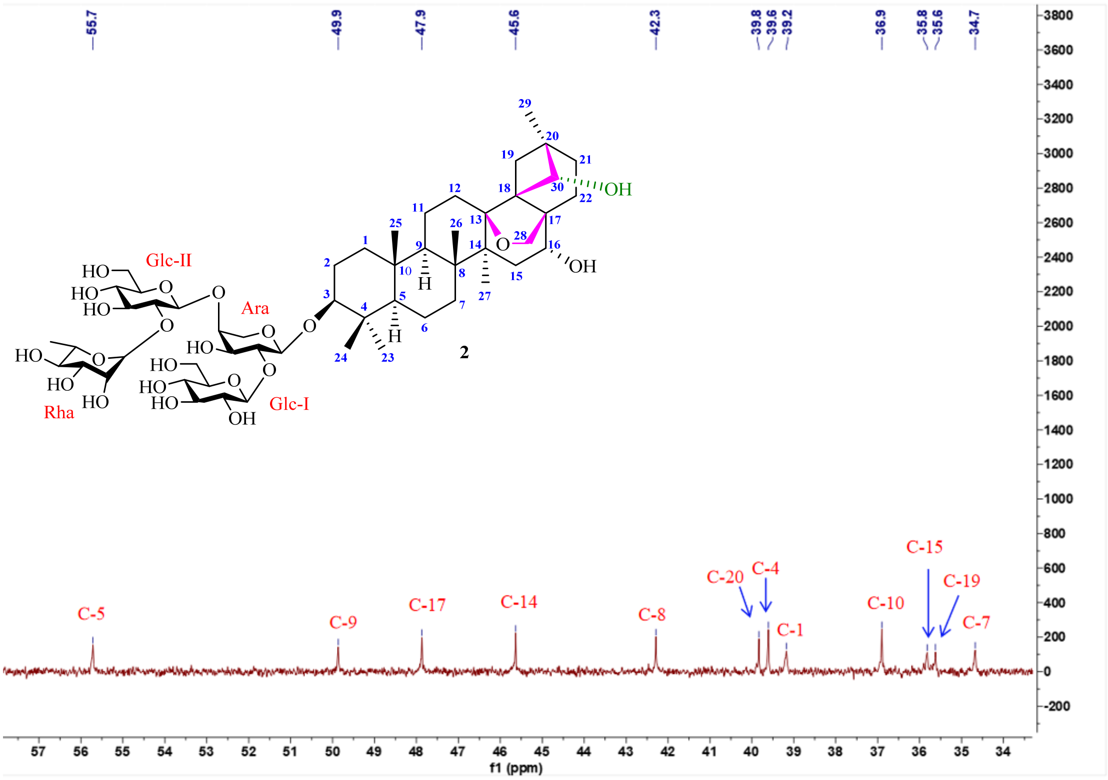

Figure S31. ^{13}C NMR assignment-2 of compound **2**

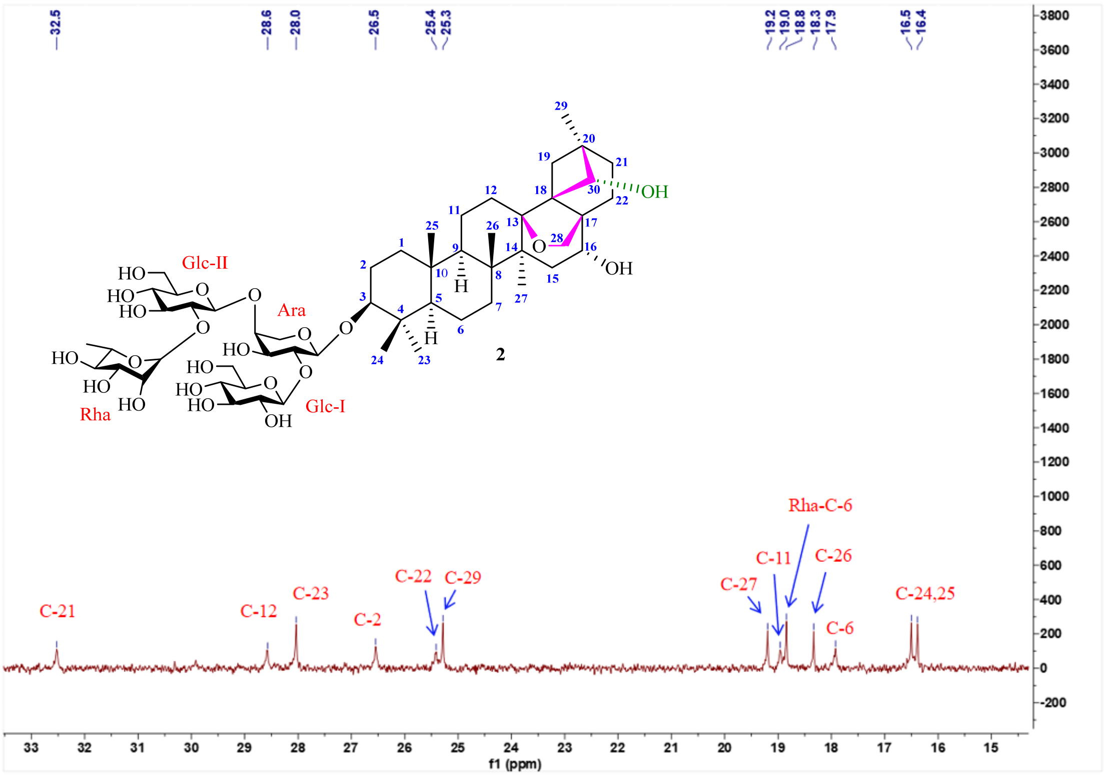

Figure S32. ^{13}C NMR assignment-3 of compound **2**

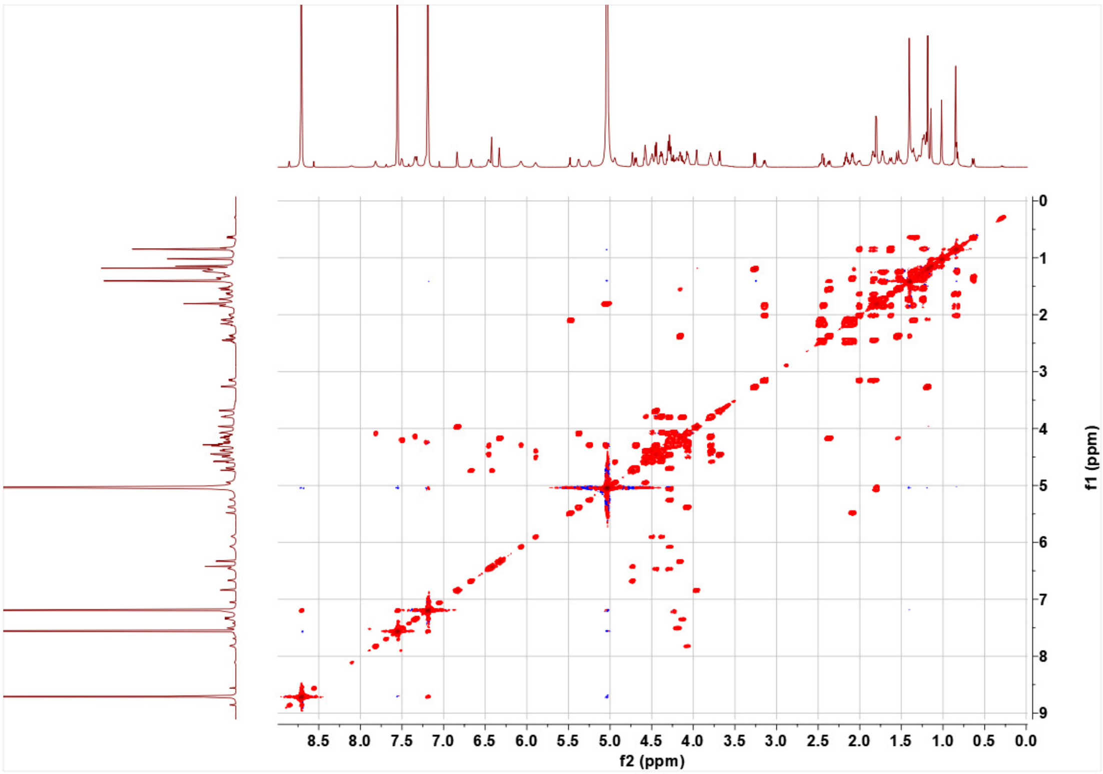

Figure S33. COSY spectrum-1 of compound **2**

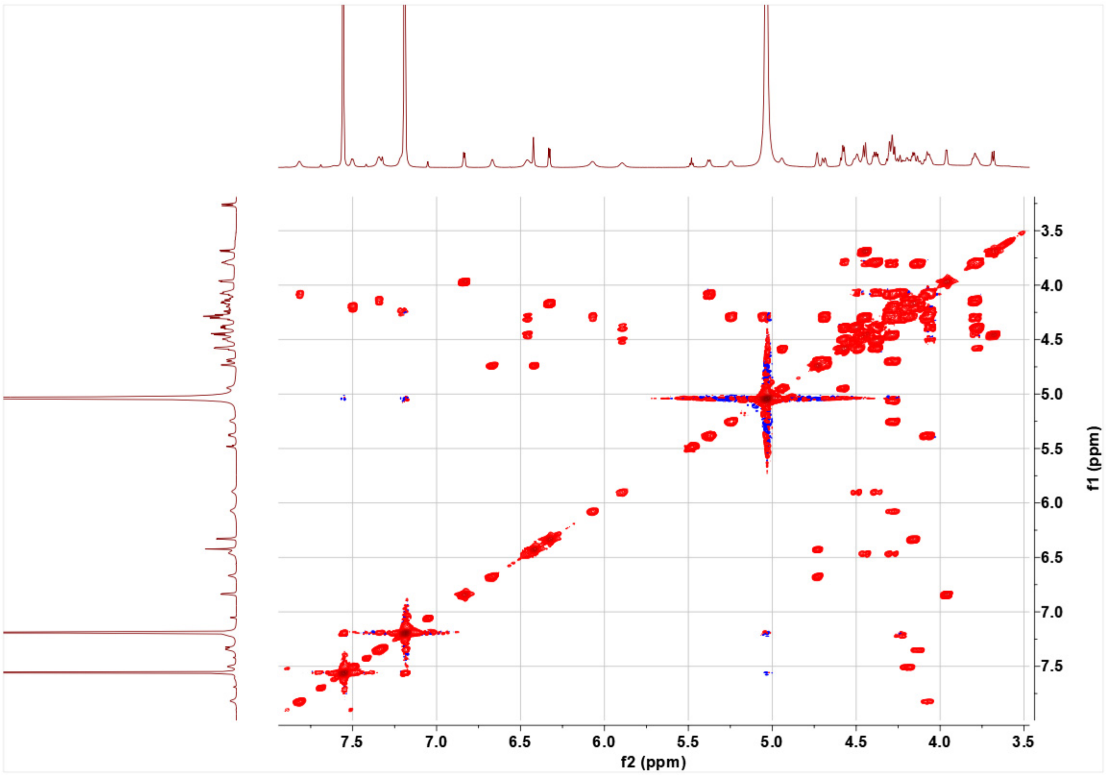

Figure S34. COSY spectrum-2 of compound **2**

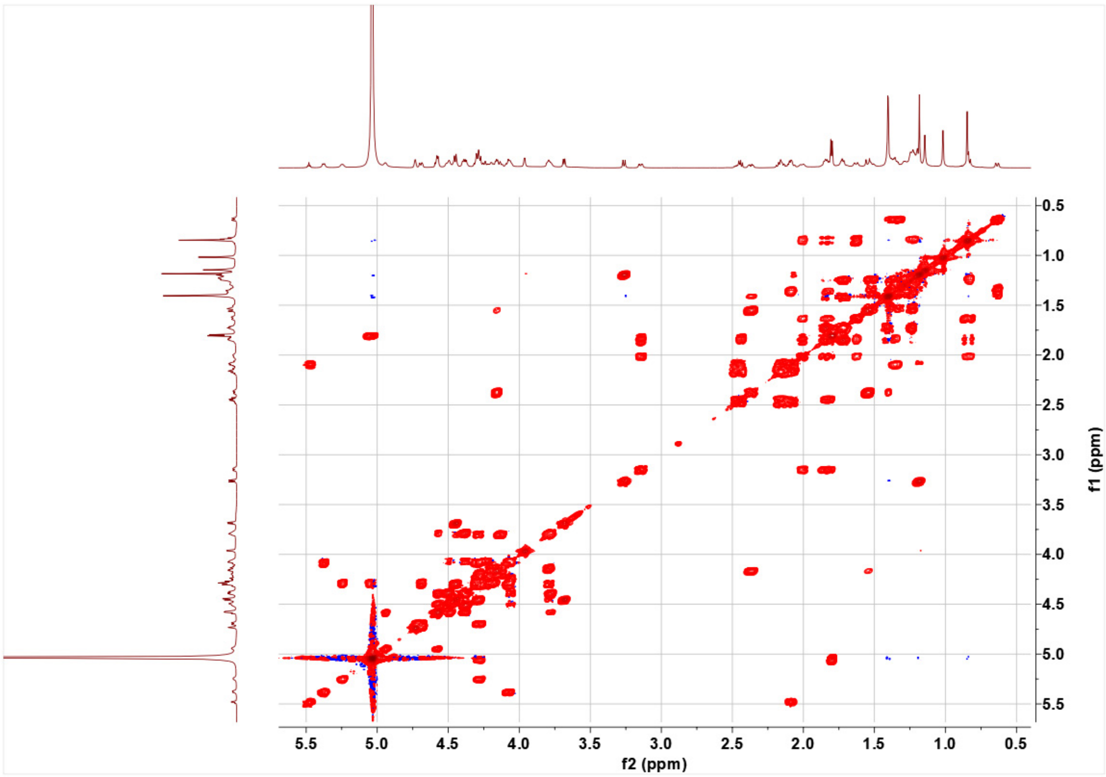

Figure S35. COSY spectrum-3 of compound **2**

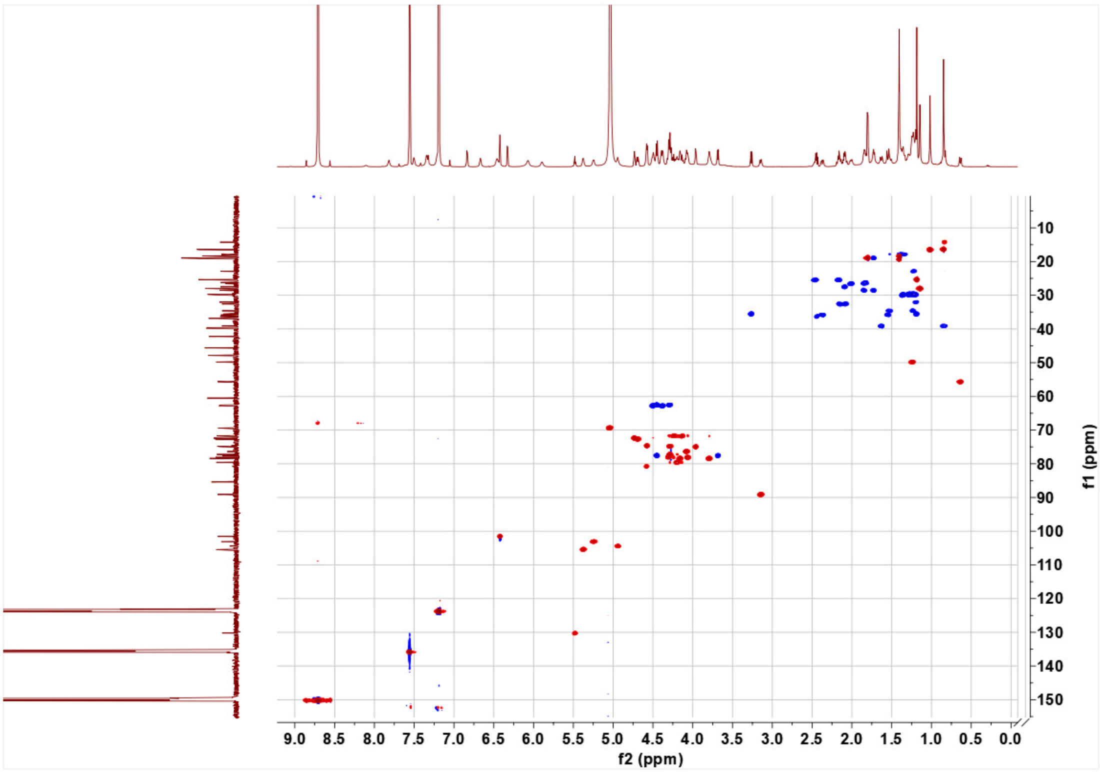

Figure S36. HSQC spectrum-1 of compound **2**

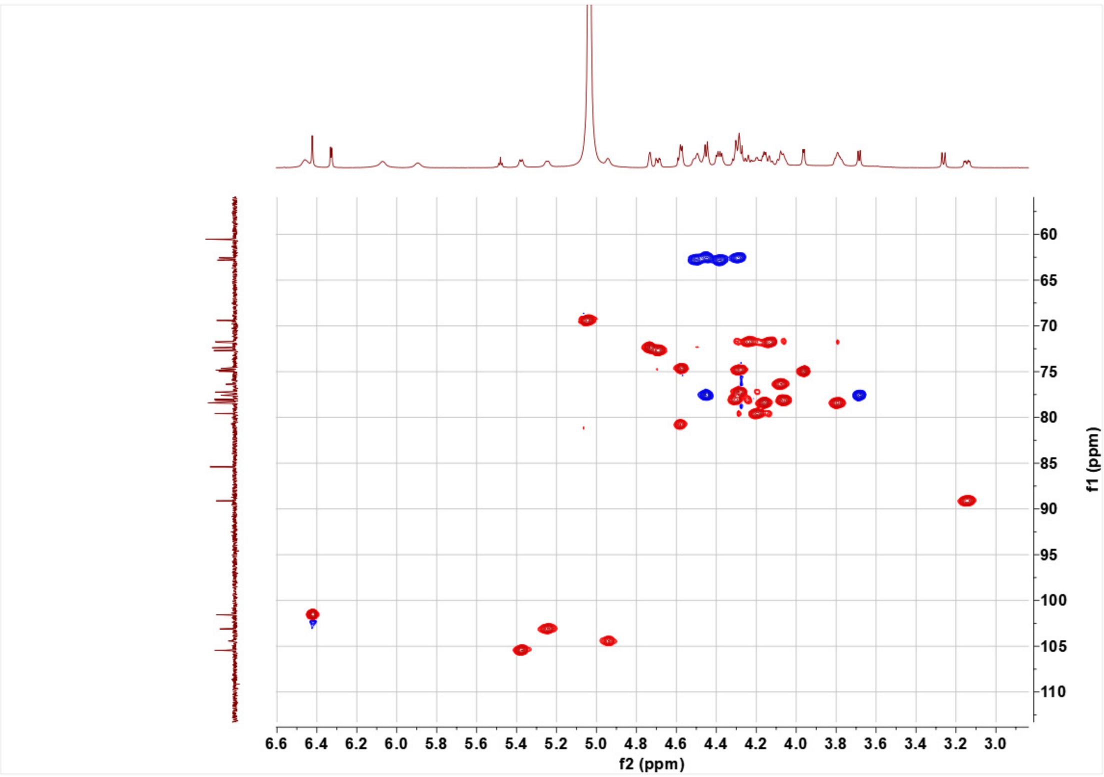

Figure S37. HSQC spectrum-2 of compound **2**

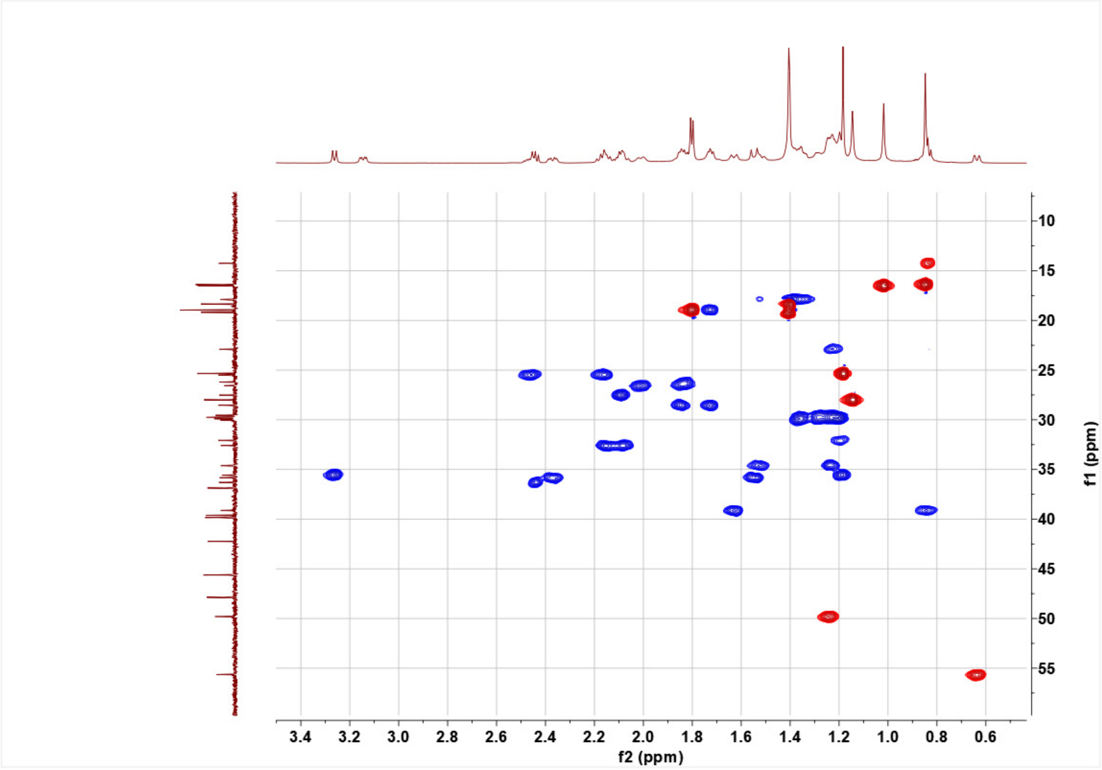

Figure S38. HSQC spectrum-3 of compound **2**

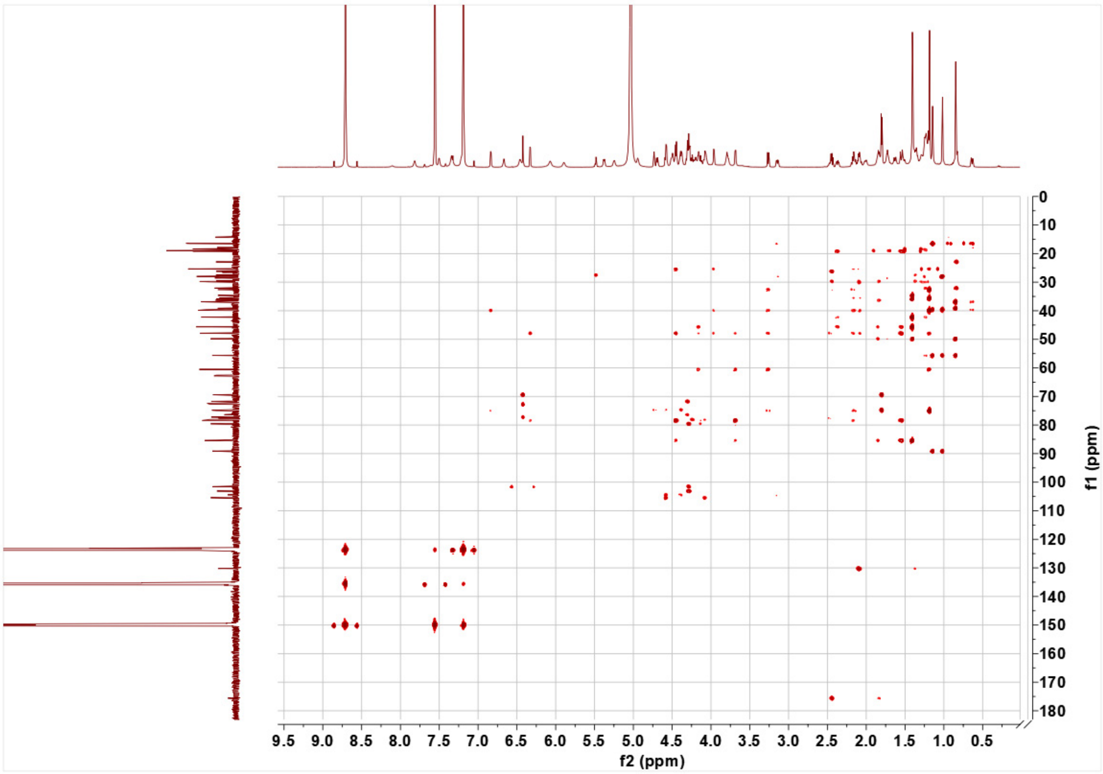

Figure S39. HMBC spectrum-1 of compound **2**

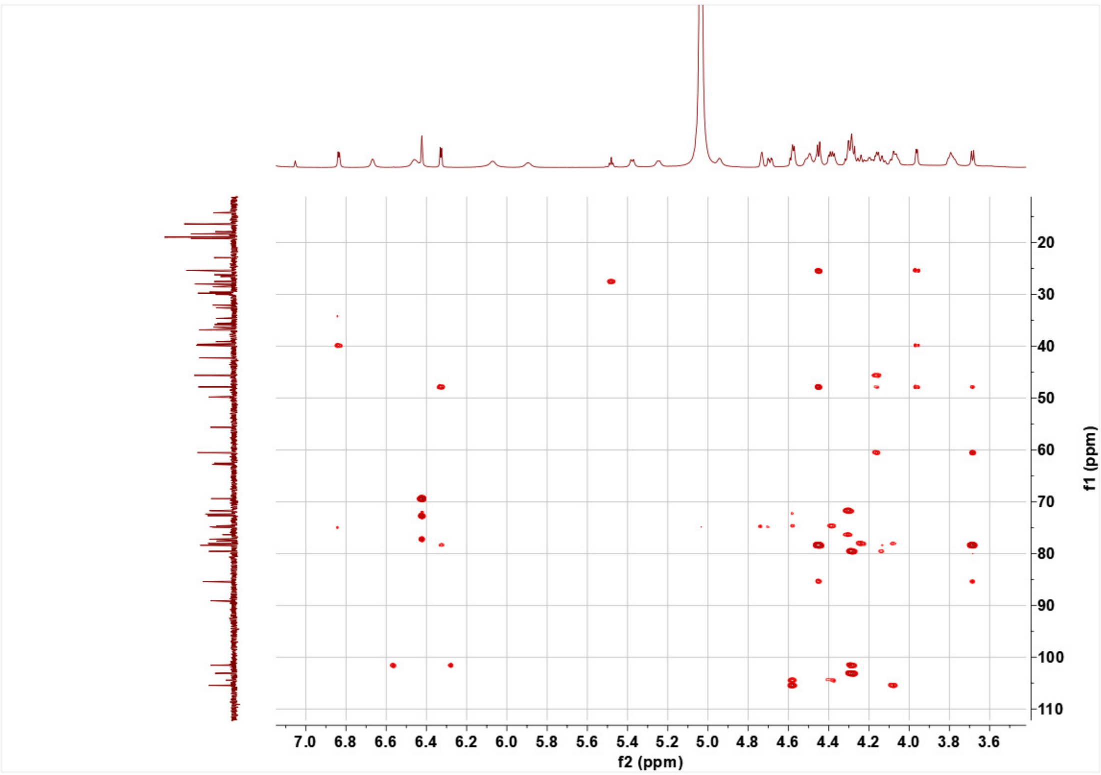

Figure S40. HMBC spectrum-2 of compound **2**

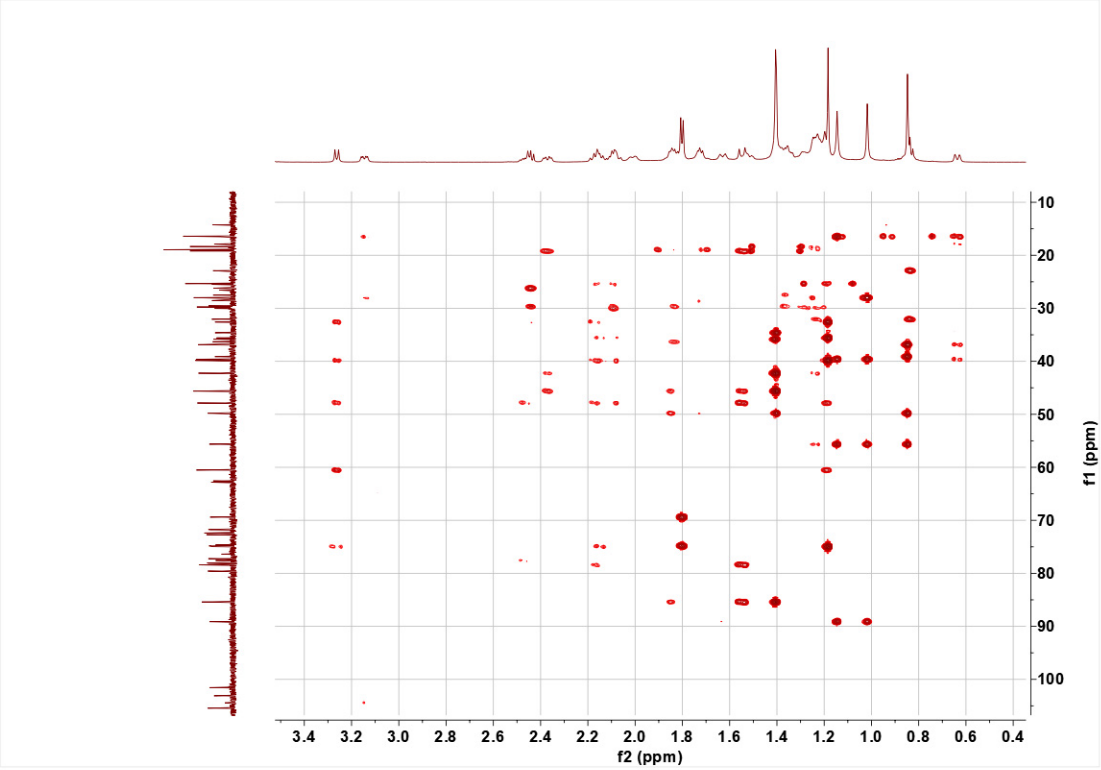

Figure S41. HMBC spectrum-3 of compound **2**

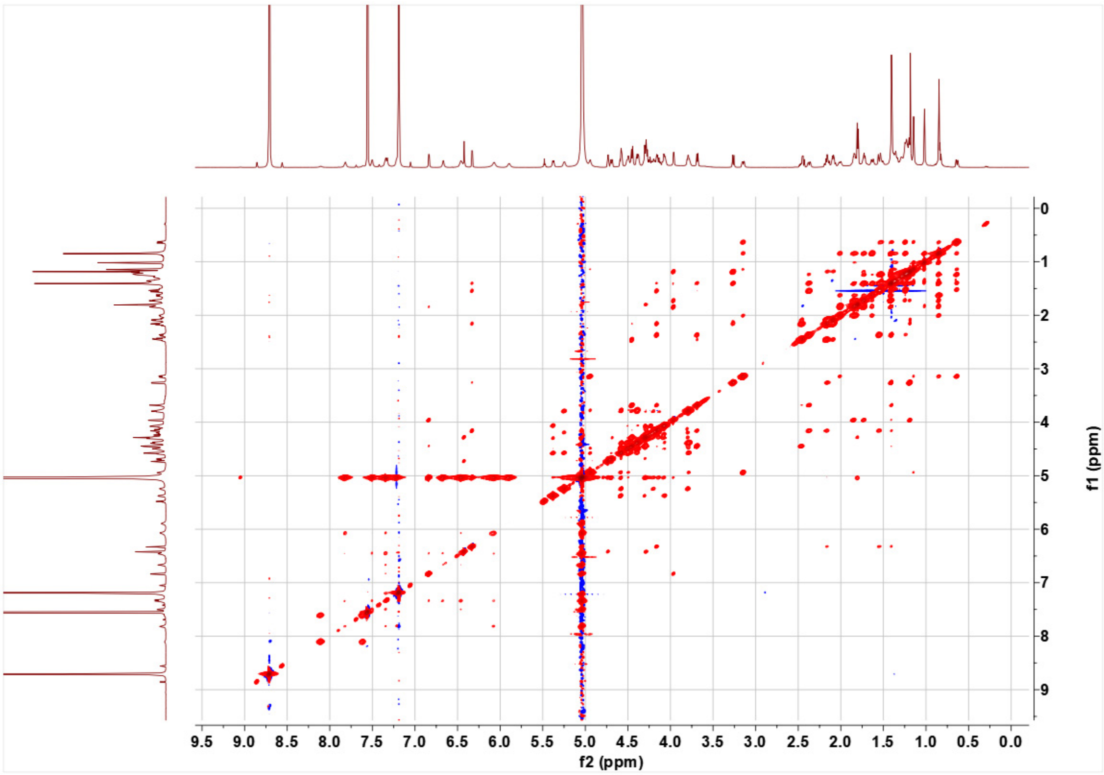

Figure S42. NOESY spectrum-1 of compound **2**

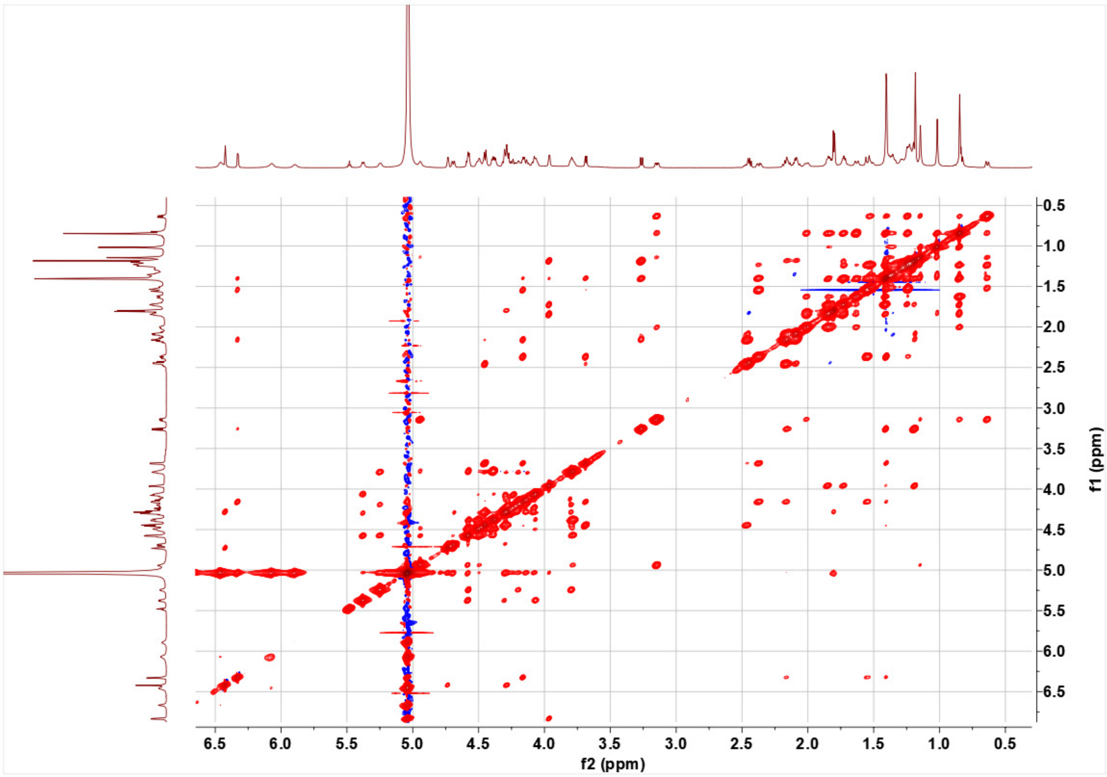

Figure S43. NOESY spectrum-2 of compound **2**

Table S1. Experimental and computed ^{13}C NMR chemical shifts of **1** and **2**

Position	Experimental	Calculated		Experimental	Calculated	
	**1**	**1**	**2**	**2**	**1**	**2**
1	39.0	38.0	38.5	39.2	37.6	38.1
2	26.5	22.5	22.5	26.5	22.6	22.4
3	89.1	85.0	86.4	89.2	83.5	85.2
4	39.5	42.3	42.8	39.6	41.8	42.4
5	55.5	54.5	55.3	55.7	53.7	54.6
6	17.9	20.0	20.0	17.9	20.1	19.9
7	34.6	33.9	34.3	34.7	33.7	34.0
8	42.5	45.4	45.8	42.3	44.8	45.3
9	49.6	49.0	50.4	49.9	48.4	49.8
10	36.7	40.1	40.7	36.9	39.7	40.3
11	19.1	20.8	20.8	19.0	20.9	20.7
12	28.9	31.6	29.6	28.6	31.4	29.4
13	88.9	88.8	86.3	85.4	87.1	85.1
14	46.1	49.2	49.4	45.6	48.6	48.8
15	35.8	35.9	36.4	35.8	35.6	36.0
16	78.0	78.9	80.5	78.4	77.5	79.4
17	49.6	51.4	50.7	47.9	50.7	50.1
18	56.8	58.6	61.3	60.5	57.7	60.5

19	41.1	39.9	34.7	35.6	39.5	34.4
20	40.3	40.9	42.5	39.8	40.5	42.1
21	36.8	36.9	33.2	32.6	36.6	32.9
22	26.0	26.6	26.5	25.4	26.6	26.3
23	28.0	25.8	25.9	28.0	25.8	25.8
24	16.4	15.5	15.4	16.5	15.7	15.4
25	16.3	15.5	15.6	16.4	15.7	15.6
26	18.3	17.2	17.3	18.3	17.3	17.3
27	19.1	17.7	17.7	19.2	17.8	17.6
28	76.4	74.1	76.7	77.5	72.8	75.7
29	21.3	19.2	23.1	25.3	19.4	23.0
30	81.0	81.0	75.7	75.0	79.5	74.6

Table S2. Experimental and computed ^{1}H NMR chemical shifts of **1** and **2**

Position	Experimental	Calculated		Experimental	Calculated	
	**1**	**1**	**2**	**2**	**1**	**2**
1a	1.59	2.00	2.06	1.63	1.93	2.01
1b	0.76	1.12	1.15	0.84	1.11	1.09
2a	1.95	2.13	2.19	2.01	2.05	2.14
2b	1.81	1.51	1.56	1.84	1.47	1.50
3	3.10	3.03	3.08	3.15	2.88	3.05
5	0.61	0.96	0.98	0.64	0.96	0.91
6a	1.38	1.66	1.71	1.38	1.61	1.66
6b	1.33	1.65	1.68	1.34	1.61	1.62
7a	1.51	1.77	1.85	1.52	1.72	1.79
7b	1.22	1.38	1.42	1.23	1.35	1.36
9	1.27	1.49	1.53	1.24	1.46	1.47
11a	1.73	1.76	1.86	1.73	1.71	1.80
11b	1.45	1.62	1.69	1.42	1.58	1.63
12a	3.08	2.55	2.13	1.85	2.44	2.08
12b	2.35	1.77	1.86	1.73	1.72	1.80
15a	2.33	2.36	2.44	2.37	2.27	2.40
15b	1.56	1.31	1.34	1.55	1.29	1.27
16	4.15	4.27	4.33	4.16	4.03	4.32
19a	3.39	2.96	2.90	3.26	2.82	2.87
19b	2.74	2.66	1.32	1.19	2.54	1.26
21a	2.38	2.16	2.07	2.15	2.08	2.02
21b	1.79	1.92	1.96	2.08	1.86	1.91
22a	1.99	1.90	2.29	2.46	1.84	2.25
22b	1.83	1.96	2.09	2.17	1.89	2.04

23a	1.13	1.13	1.16	1.15	1.12	1.10
23b	1.13	1.13	1.16	1.15	1.12	1.10
23c	1.13	1.13	1.16	1.15	1.12	1.10
24a	0.99	0.88	0.93	1.02	0.89	0.86
24b	0.99	0.88	0.93	1.02	0.89	0.86
24c	0.99	0.88	0.93	1.02	0.89	0.86
25a	0.80	0.99	1.06	0.85	1.00	0.99
25b	0.80	0.99	1.06	0.85	1.00	0.99
25c	0.80	0.99	1.06	0.85	1.00	0.99
26a	1.38	1.28	1.34	1.40	1.27	1.27
26b	1.38	1.28	1.34	1.40	1.27	1.27
26c	1.38	1.28	1.34	1.40	1.27	1.27
27a	1.52	1.34	1.34	1.41	1.32	1.27
27b	1.52	1.34	1.34	1.41	1.32	1.27
27c	1.52	1.34	1.34	1.41	1.32	1.27
28a	3.84	3.86	4.21	4.45	3.65	4.20
28b	3.70	3.82	3.79	3.68	3.62	3.77
29a	1.24	1.13	1.18	1.18	1.12	1.11
29b	1.24	1.13	1.18	1.18	1.12	1.11
29c	1.24	1.13	1.18	1.18	1.12	1.11
30	4.56	4.72	4.07	3.96	4.45	4.06

Table S3. Statistics of ordinary least squares (OLS) linear regression of experimental and computed ^{13}C and ^{1}H NMR chemical shifts of **1** and **2**

Experimental	Type	Compd.	CMAD[a]	CLAD[b]	R^2	R^2_{adj}	RMSE	F	p value
**1**	^{13}C	**1**	1.6	4.2	0.9917	0.9914	2.0	3359.48	< 0.01
		**2**	2.2	6.4	0.9845	0.9840	2.8	1780.08	< 0.01
	^{1}H	**1**	0.18	0.58	0.9451	0.9438	0.23	739.72	< 0.01
		**2**	0.25	1.42	0.8604	0.8571	0.36	264.95	< 0.01
**2**	^{13}C	**1**	2.4	6.1	0.9818	0.9812	2.94	1511.82	< 0.01
		**2**	1.6	4.2	0.9914	0.9911	2.02	3231.96	< 0.01
	^{1}H	**1**	0.22	1.35	0.8719	0.8689	0.33	292.64	< 0.01
		**2**	0.16	0.39	0.9588	0.9578	0.19	1000.33	< 0.01

[a] CMAD = corrected mean absolute deviation, computed as $(1/n)\sum_{i}^{n}|\delta_{calc}-\delta_{exp}|$, where δ_{calc} and δ_{exp} refer to the calculated and experimental chemical shifts. [b] CLAD = corrected largest absolute deviation, computed as $\max(|\delta_{calc}-\delta_{exp}|)$.

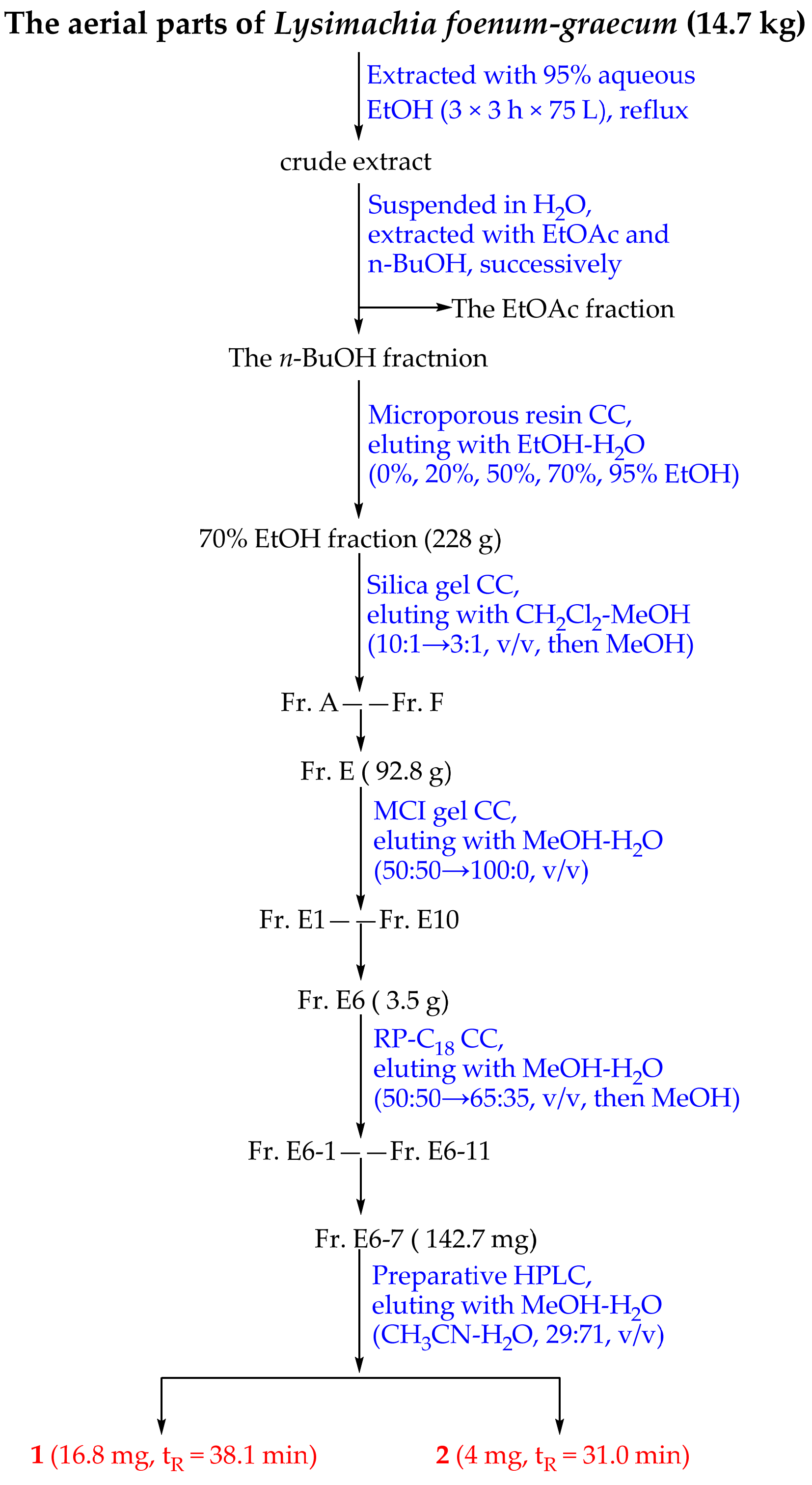

Figure S44. Isolation and purification of compounds **1** and **2**
